# The Role of *In Situ*/*Operando* IR Spectroscopy in Unraveling Adsorbate-Induced Structural Changes in Heterogeneous Catalysis

**DOI:** 10.1021/acs.chemrev.3c00372

**Published:** 2023-10-26

**Authors:** Elena Groppo, Sergio Rojas-Buzo, Silvia Bordiga

**Affiliations:** Department of Chemistry, NIS Centre and INSTM, University of Torino, via Giuria 7, 10125 Turin, Italy

## Abstract

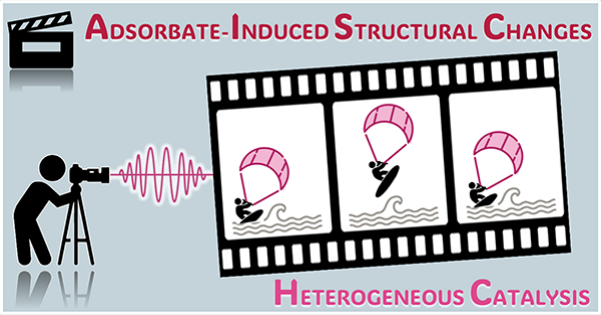

Heterogeneous catalysts undergo thermal- and/or adsorbate-induced dynamic changes under reaction conditions, which consequently modify their catalytic behavior. Hence, it is increasingly crucial to characterize the properties of a catalyst under reaction conditions through the so-called “*operando*” approach. *Operando* IR spectroscopy is probably one of the most ubiquitous and versatile characterization methods in the field of heterogeneous catalysis, but its potential in identifying adsorbate- and thermal-induced phenomena is often overlooked in favor of other less accessible methods, such as XAS spectroscopy and high-resolution microscopy. Without detracting from these techniques, and while aware of the enormous value of a multitechnique approach, the purpose of this Review is to show that IR spectroscopy *alone* can provide relevant information in this field. This is done by discussing a few selected case studies from our own research experience, which belong to the categories of both “single-site”- and nanoparticle-based catalysts.

## 1 Introduction

The definition of a heterogeneous catalyst as a material that increases the rate of a chemical reaction without itself undergoing any permanent chemical change might give the false impression that a heterogeneous catalyst is a static material. Actually this is not the case, and any researcher involved in the field of heterogeneous catalysis knows that the surface and bulk structure of a heterogeneous catalyst dynamically change in the presence of reactants, intermediates, and products.^1−4^ The number of works reporting on the occurrence of adsorbate-induced structural changes in heterogeneous catalysts under reaction conditions is nowadays uncountable, fueled by the incredible advances in the characterization methods, which made it possible to detect dynamic phenomena occurring at increasingly faster time scales and with ever-increasing spatial resolution.

In this respect, catalysts based on supported metal nanoparticles (NPs) are by far the most investigated systems. A first example of adsorbate-induced structural change involving supported metal nanoparticles is their disruption. This phenomenon was demonstrated to occur more than 50 years ago on Rh NPs in the presence of carbon monoxide, leading to volatile rhodium carbonyls that can redeposit at the surface of the support.^5^ Sintering is the opposite phenomenon, *i.e.*, the agglomeration and gradual growth of metal NPs, which is often triggered by temperature and in some cases mediated by the adsorbates. It constitutes one of the most common causes of catalyst deactivation^6−9^ and can be at least partially contrasted by “redispersion” in an oxidative environment, which is a common practice in the petrochemical industry. Adsorbates may also induce the reconstruction of a metal surface. Evidence of the occurrence of adsorbate-induced surface reconstruction on metal single-crystals date back to the 1960s, when ultrahigh vacuum (UHV) technology became widely available.^10^ For metal NPs, where the border between surface and bulk is poorly defined, adsorbate-induced restructuring might involve whole particles; in this case, a change in morphology might even occur. Adsorbates might also induce the segregation of bimetallic NPs, promoting the formation of core–shell structures. These examples (and many others reported in the literature) show that supported metal NPs are not passive and immutable entities; rather, they interact with adsorbates (reactants, intermediates or products) in a dynamic and flexible way, undergoing a series of surface and structural changes that can ultimately influence their catalytic behavior.^2,11−17^

Adsorbate-induced structural changes are not a prerogative of catalysts based on supported metal NPs. Single-site heterogeneous catalysts also experience similar phenomena, even though they are much less recognized. Generally speaking, adsorbates cause an expansion of the coordination sphere around the metal active sites, which, as a consequence, rearrange at the surface of the support. Structural rearrangement might involve changes in bond distances and angles, which indicate that the support itself has a certain flexibility, up to a full solvation and mobilization of the active sites by the adsorbate, as demonstrated to occur, for example, in Cu-zeolites in the presence of ammonia.^18−22^

It is clear that such changes at the atomic level, which are an inherent part of the catalyst’s identity, induce modifications of the chemical properties and have an effect on the catalytic performance as a whole. This explains why it is increasingly crucial to characterize the surface and bulk properties of a catalyst under reaction conditions as close as possible to those experienced by the catalysts into the reactor, simultaneously collecting activity and selectivity data, which is the so-called “*operando*” approach.^23−26^ In the last few decades, strong improvements in the characterization techniques as well as in the associated experimental set-ups have been achieved, allowing the investigation of heterogeneous catalysts under almost any reaction conditions, with an unprecedented time and spatial resolution. These progresses involve both spectroscopic methods and microscopic tools. Nowadays, there exist many reactor cells, allowing spectroscopy (spanning from X-rays to IR) to be performed under reaction conditions very close to industrial ones.^27−29^ Additionally, surface sensitive spectroscopies such as XPS are no longer restricted to UHV environments and can be performed at elevated pressures or even in wet/liquid atmospheres.^30−32^ Modern electron microscopies allow work in the presence of gases (even at atmospheric pressure) reaching atomic resolution,^33^ while X-ray microscopies allow the collection of spectroscopic information with a spatial resolution in the micrometer to nanometer scale.

Among all the cited techniques, IR spectroscopy is probably the most ubiquitous in contemporary chemical laboratories. For this reason, it is one of the most exploited methods for the *in situ*/*operando* characterization of heterogeneous catalysts.^34−36^ In fact, its simplicity and versatility make it easy to combine with a variety of other techniques, such as mass spectrometry or gas chromatography, for the on-line evaluation of the catalytic performance, often coupled with a modulation–excitation approach.^26,37−46^ Despite its popularity, its potential to unravel adsorbate-induced structural changes in heterogeneous catalysis is still not fully explored. The purpose of this Review is to highlight these potentialities, both in single-site systems and in supported nanoparticle-based catalysts. To this aim, we have selected three case studies taken from our own research experience, namely, Cr-based polymerization catalysts, metal-exchanged zeolites, and supported precious metal catalysts. It is important to notice that we entirely focus on the role of IR spectroscopy in detecting adsorbate-induced phenomena, even though in most of the selected case studies the use of complementary characterization methods was fundamental to confirm the scenario. Ideally, the data collected in the following chapters should help researchers gain awareness of the fact that a relatively simple and widely accessible characterization method can allow a rapid identification of adsorbate-induced structural changes in heterogeneous catalysis, without the need to resort to much more expensive techniques or to methods requiring access to large-scale facilities.

The structure of the Review is organized as follows. Section 2 is entirely dedicated to the Cr/SiO_2_ Phillips catalyst for ethylene polymerization, which is a protype for single-site heterogeneous catalysts. A series of results, both experimental and theoretical, reported in the literature converge to a picture where the chromium sites are not rigidly anchored to the silica surface but rather display a flexible behavior in the presence of adsorbates, mediated by surface siloxane ligands that behave literally as the ancillary ligands in homogeneous catalysis. The catalytic performances of the Phillips catalyst are revisited in terms of surface strain, and it is shown that IR spectroscopy is one of the most sensitive methods, if not the most, to spectroscopically discriminate among different chromium sites, overcoming the difficulties associated with the amorphous nature of the support and the very low chromium loading. Section 3 collects a series of examples belonging to the field of metal-substituted zeolites. It is demonstrated that the vibrational fingerprints of the zeolite framework are perturbed by the presence of heteroatoms at a different extent, depending on the zeolite topology, the Si/Al ratio, and the type of metal, its loading, and oxidation state. Although crystalline and thermally stable, zeolites are anything but rigid materials; rather, they are structurally flexible, especially in the presence of adsorbates, even at moderate temperatures. IR spectroscopy offers a powerful and relatively simple method to track the dynamic changes occurring at the metal centers in zeolites. Finally, section 4 is dedicated to catalysts based on supported Pt nanoparticles and single atoms. In this field, adsorbate-induced structural modifications and related phenomena have been increasingly reported over the last few decades. X-ray absorption spectroscopy complemented by theoretical calculations, and more recently X-ray total scattering (coupled with PDF approach) and high-resolution electron microscopy, has been so far the most employed method to demonstrate the occurrence of a change in the structure and morphology of metal nanoparticles under reaction conditions. The series of data collected in this section demonstrate that IR spectroscopy can be an alternative, widely available, and cheaper method to unravel the ductile behavior of Pt nanoparticles and single atoms in the presence of adsorbates. Moreover, IR spectroscopy allows the speciation and quantification of the metal surface sites, which are not possible with previously mentioned techniques.

## 2 The Cr/SiO_2_ Phillips Catalyst for the Polymerization of Ethylene: The Chromium Sites Are Not Rigidly Anchored to the Silica Surface

### 2.1 Single Sites or Multisites?

The Phillips catalyst for ethylene polymerization, Cr/SiO_2_, is perhaps one of the most notable examples of single-site catalysts. At present, it accounts for almost half of the global market for high-density polyethylene (HDPE) production.^47^ The precursors of the active sites are originated from a silica-supported chromium salt upon calcination at temperatures above 600 °C (also called the activation). If done correctly, this procedure can bind each chromium atom individually to the silica support in the hexavalent form, Cr(VI), from which derives the definition of a single-site catalyst.^48−50^ The active sites are then obtained from Cr(VI) in the presence of ethylene itself, which acts simultaneously as a reducing and self-alkylating agent. The self-alkylation mechanism and the molecular structure of these reduced chromium sites are still objects of controversy.^51^ Nevertheless, what is widely recognized is that the anchored chromium sites are not all the same. What can be interpreted as a defect actually turns into a merit. In fact, the presence of several types of Cr(VI) sites leads to the formation of a variety of active species with distinct kinetics in chain propagation and transfer, which produce polymer chains with various lengths. In turns, this explains the uniqueness of the Phillips HDPE products, which are characterized by a broad and adjustable molecular weight (MW) distribution.^48−50,52−57^ A broad molecular weight distribution is extremely important in determining the molding behavior and the final physical properties of the finished polyethylene, distinguishing the Phillips catalyst from all the other catalysts industrially employed for polyethylene production.

There are several reasons that explain why the anchored chromium sites are not all the same. The first source of site heterogeneity is the degree of polymerization of the chromate species (monochromate, dichromate, and even polychromates). This subject has been at the center of scientific discussion for some decades.^48−51,58−61^ For catalysts with a low chromium loading (below 1 wt %), most of the experimental evidence points toward the exclusive presence of monochromate species, while at higher metal loadings diffuse reflectance UV–vis and Raman spectroscopies support the presence of dichromates.^59,62−84^ Beside the degree of polymerization of the chromate species, another source of heterogeneity is represented by the anchoring mode of Cr(VI). Focusing on the monomeric Cr(VI) species, the doubly anchored tetrahedral dioxo (SiO)_2_Cr(=O)_2_ unit is by far the most frequently encountered, but four-coordinate pentahedral monooxo (SiO)_4_—Cr=O structures may also occur as a minority species when four silanol sites are suitably arranged. Spectroscopic evidence for the presence of both types of sites has been reported in the literature.^59,62−84^

However, the fundamental source of site multiplicity is the possibility for the chromium sites to adopt different conformations in terms of strain and local environment as a consequence of the amorphous nature of the silica support featuring abundant silanol groups. Indeed, on the silica surface there is a wide range of Si–Si distances between the silanol pairs used for anchoring the dioxo chromium structures.^85−93^ This leads to variations in the O–Cr–O angle and Cr–O–Si bond distances. To complicate the scenario, it has been demonstrated that Cr(VI) species are highly mobile at the silica surface and can move not only within the silica particle but also between one particle and another during the activation step.^48,55^ This not only increases the possibilities to exploit silanol pairs characterized by long Si–Si distances for anchoring but might also allow the anchoring of the chromium sites by the breakage of the siloxane bridges resulting from surface dehydroxylation.

### 2.2 Active-Site Strain and Reactivity: An Inseparable Couple

Manipulation of the activation procedure is one of the simplest and most efficient approaches to tailor the Si–Si distance between silanol pairs, with relevant consequences on both the catalytic activity and properties of the obtained polymer.^55^ Even though esterification of CrO_3_ with the surface silanol groups occurs at a relatively low temperature (150–350 °C), the Phillips catalyst needs to be calcined at temperatures above 500 °C to show appreciable polymerization activity, and the activity raises with the increase in the activation temperature. The strict relationship between the calcination temperature and the catalytic activity was traditionally explained on account of two main phenomena. First, increasing the activation temperature induces the progressive condensation of proximal silanol groups, which become more isolated. A decrease in the amount of silanol groups is beneficial for the catalytic activity, since silanols increase the steric hindrance around the chromium active sites and hence act as poisons. Second, condensation of the silanol groups is accompanied by the formation of siloxane rings with smaller sizes (and water as byproduct), and as a consequence the conformation of the anchored Cr(VI) species changes. Chromium sites anchored onto smaller siloxane rings should experience a higher strain, and this would explain why the calcination temperature strongly affects the catalytic activity and the polymer properties. In particular, by increasing the calcination temperature, a lower polymer MW, narrower MW distribution, and increased comonomer incorporation can be achieved.

A correlation between strain at metal sites and reactivity in silica-supported metal catalysts was proposed also for other systems. For example, Amakawa et al.^94^ ascribed the remarkable increase in the reactivity of Mo/SiO_2_ at high molybdenum loadings to an increased frustration of the surface molybdenum oxide species. Limited availability of anchoring silanol groups at high molybdenum loadings forces the MoO_4_ moieties to occupy more strained configurations, which correspond to a higher reactivity. For the Phillips catalyst, McDaniel proposed an intuitive idea,^48−50,55^ not supported by any experimental data, according to which the Cr–O–Si strain would tend to pull the chromium sites away from the shared oxide orbitals, enhancing their positive charge (*i.e.*, the electron deficiency, or Lewis acidity) and hence their activity. This concept was addressed by computation in the early 2000s by Espelid and Borve,^95−98^ who performed a series of systematic DFT investigations on small clusters representative of the “naked” Cr(II) sites in the Phillips catalyst and found that the active site strain decreases when the size of the chromasiloxane rings expanded. They first proposed a six-membered chromasiloxane ring (6CR) as a key model of the active site on the Phillips chromium catalyst. Several other computational studies have focused on the role of strain in affecting the activity of the Phillips catalyst in ethylene polymerization, and some of them were also able to reproduce some experimental data.^99,100^

**Figure 1:**
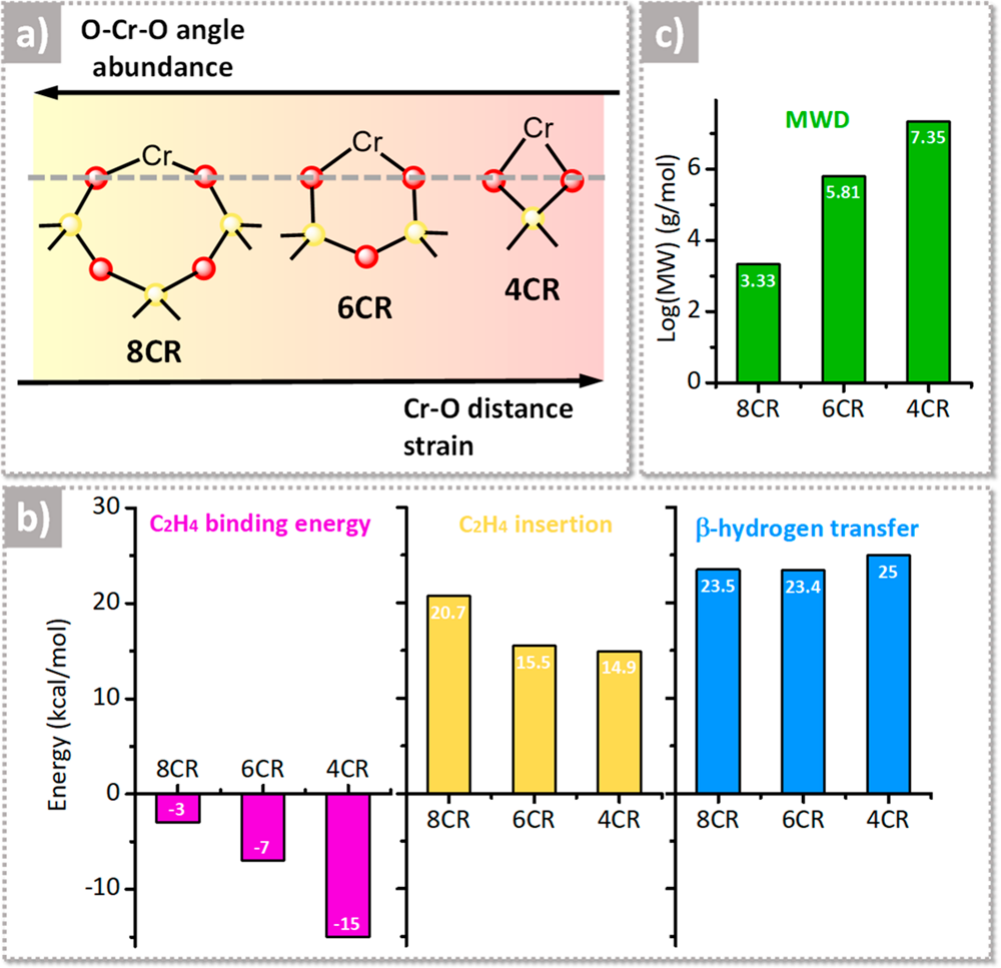
Relationship between the strain of the active chromium sites and reactivity, as determined by the computational work in ref (103). (a) Simplified illustration of the four-, six-, and eight-membered chromasiloxane ring (4CR, 6CR, and 8CR, respectively) models bearing Cr(II) species, with a qualitative indication of their relative abundance and strain, and the correlation with the Cr–O distance and O–Cr–O angle. By moving from yellow to red, the strain increases. (b) Most relevant energetic values as extrapolated from the Gibbs free energy profiles for ethylene binding on Cr(II) and for ethylene insertion and β-hydrogen transfer on the corresponding Cr(III)-ethyl models. (c) Predicted average MW of the PE obtained from the three models, determined by the kinetic competition between chain propagation and chain transfer. Data reproduced with permission from ref (103). Copyright 2022 American Chemical Society.

Recently, Floryan et al.^101^ proposed to use the Cr–O bond length as a descriptor for active site strain. For a series of Cr(III)/SiO_2_ models, all of them based on a Cr(III) ion bonded to the silica surface via three siloxy linkages, they predicted that sites with longer Cr–O bonds and smaller O–Cr–O angles were more strained and exhibited much faster initiation and propagation kinetics in ethylene polymerization. The same results were obtained for Cr(III) sites on a periodic model of silica, irrespective of the initiation mechanism (via ethylene insertion into the Cr–O bond or via C–H activation).^102^ The most recent complete computational work that addressed the issue of active site strain and catalytic performances was reported by Huang et al.^103^ on a large number of cluster models, comprising four-, six-, and eight-membered chromasiloxane rings (4CR, 6CR, and 8CR, respectively). The main results are summarized in a simplified way in Figure 1a, where the focus is placed also on the distance of the chromium species from the surface defined by the two covalently bonded oxygen atoms. For Cr(VI) models, in agreement with previously mentioned works, 4CR is characterized by longer Cr–O bonds and much smaller O–Cr–O angles, hence by a greater strain. The positive charge for 4CR is almost the same as for 6CR and higher than for 8CR, as was suggested intuitively by McDaniel.^48−50,55^ According to the formation energies, the CR size availability should increase when the size of CR increases, making the 4CR species the least abundant. The same trend is predicted for the Cr(II) models.

Figure 1b summarizes the most relevant energetic values as extrapolated from the Gibbs free energy profiles for ethylene binding on Cr(II) and for insertion and β-hydrogen transfer on the corresponding Cr(III)–ethyl models. The values of the binding energies for two ethylene molecules on each Cr(II) model are inversely proportional to the O–Cr–O angle, which suggests that steric effects play an important role in ethylene binding: the more open is the site, the easier is the binding. As far as the energy barriers for ethylene insertion into the Cr(III)–ethyl bond are concerned, 4CR and 6CR have insertion barriers approximately 5 kcal/mol lower than that for 8CR, suggesting a slower chain initiation process on the latter. In contrast, the energy barrier for the β-hydrogen transfer is almost independent of the CR size. As a consequence, the three models are predicted to produce PE with a different MW, which is determined by the kinetic competition between chain propagation and chain transfer (Figure 1c). The model with smaller CR, which is the most strained, is predicted to produce a PE with an ultrahigh MW, which is centered at 7.35 g/mol in log values. In contrast, the 8CR model, which is the least strained, is expected to produce a PE with a low MW (3.33 g/mol in log values), while the model with intermediate strain should produce a PE with an intermediate MW (5.81 g/mol in log values).

Even though the results summarized above look consistent and solid, it is important to remember that the structural parameters determined by calculation are very much dependent on the model and on the level of calculation. For example, in late 2009, Damin et al.^104^ performed a systematic computational investigation on very simple X_4_Si_2_O_3_Cr (X = H, OH, F) clusters (the same used by Espelid and Borve in their seminal works)^95−98^ and demonstrated that the optimized geometries and the positive charge on the Cr(II) sites are strongly influenced by both the adopted functional and the cluster capping group (X). On this basis, it should not come as a surprise that different geometrical parameters have been obtained when adopting different models and/or different level of calculation. For example, Stiegman and co-workers adopted very simple clusters with Cr(VI) belonging either to a strained 6CR or to a larger and less strained 10CR, the latter derived from another structure previously used by Dines and Inglis.^80^ While the O–Cr–O angle follows the same trend found by Huang et al.,^103^*i.e.*, the angle increases with increasing the CR size, the Cr–O bond distance does not, being larger for the 10CR model (1.753 vs 1.746 Å). For Cr(II) systems, Fong et al.^105,106^ found very similar Cr–O distances (1.82 Å) for 6CR and 8CR models largely differing in the O–Cr–O angles (110° vs 132°, respectively), while for a 6CR model comprising two additional siloxane bridges in the chromium coordination sphere they found a relevant elongation of the Cr–O distance (1.87 Å) while the O–Cr–O angle was kept almost constant.

A similar situation has been reported by Budnick et al.^107^ for larger Cr(II) models, which were treated with the ONIOM embedding approach (*i.e.*, modeling the most relevant portion of the model around the chromium center at a high level of theory, adopting quantum mechanics, and the remaining portion at a lower level of theory). In particular, the authors concentrated on two models having formula H_6_O_48_Si_22_Cr (*i.e.*, almost three times larger than those adopted by Huang et al.,^103^). In the first one, Cr(II) species are saturating two distal Si–O dangling bonds belonging to a five-membered SiO_4_ ring, generating a 6CR (*vide infra* model **3** in Figure 4b), while in the second, the Cr(II) species are part of a 8CR (*vide infra* model **4** in Figure 4b). In this case, the 8CR model has Cr–O distances much longer than the 6CR one (1.938 vs 1.888 Å), despite being characterized by a very large O–Cr–O angle (161.2° vs 111.9°, respectively).

Even though this literature survey is surely not exhaustive, it allows us to make a few comments. Both the choice of the model and the level of calculation have a strong impact on the structural parameters characterizing the chromium site. While the O–Cr–O angle always correlates with the size of the chromasiloxane ring, the Cr–O distance does not. On this basis, it is recommended to use the O–Cr–O angle, and not the Cr–O bond length as suggested by Floryan et al.,^101^ as a descriptor for active site strain. The narrower the O–Cr–O angle, the more strained the chromium site. By doing so, it is probably possible to reconcile most of the computational works addressing the issue of the strain of chromium sites at the silica surface.

### 2.3 Is It Possible to Spectroscopically Discriminate the Cr Sites as a Function of Their Strain? The Unique Role Played by IR Spectroscopy

The computational results summarized above qualitatively describe the reason why a PE obtained with a Cr/SiO_2_ catalyst activated at moderate temperature shows a broad MW distribution and are also able to explain the experimental observations about the effect of the calcination temperature on both the polymerization rate and the MW distribution of the obtained polymer. The coexistence of chromium sites characterized by different conformations is a clue of their adaptability to multiple environmental situations. While being the key for the industrial success of the Phillips catalyst, this adaptability undoubtedly represents a challenge in terms of characterization. Having understood that there is a clear correlation between active site strain and catalytic performance, a significant step forward would consist in the ability to differentiate experimentally chromium sites characterized by a different strain. However, there are no experimental methods able to quantitatively evaluate bond distances and angles in this case. X-ray diffraction fails in the presence of amorphous systems and a PDF approach is also probably powerless, considering the very small amount of chromium in these systems. EXAFS spectroscopy, which in principle is able to provide geometrical details at the atomic scale also for very diluted samples, can at best provide average bond distances, without distinguishing between different chromium conformations coexisting at the silica surface.

Some information is potentially obtainable by means of UV–vis spectroscopy. In fact, we have seen that the strain of the Si–O–Cr bond affects the electron density at the chromium sites and hence their electronic properties. As a matter of fact, recent TD-DFT calculation by Stiegman and co-workers^84^ demonstrated that distinguishable differences are observed in the simulated UV–vis spectra for monomeric Cr(VI) sites grafted on strained siloxane rings (a 6CR model, using the nomenclature above) or on larger (less strained) siloxane rings (a 10 R model). The problem is that the heterogeneity of Cr(VI) sites in real samples causes a broadening of all the spectral bands, making actually impossible to figure out the relative abundance of less and more strained strained Cr(VI) species. Similar and even greater problems are encountered with XANES spectroscopy.^51,108^

Here it is where IR spectroscopy can make the difference, at least when dealing with the Cr/SiO_2_ catalyst in its CO-reduced state, which is the situation in which the adaptability of the chromium sites at the silica surface is expressed at the maximum level.^51,59−61^ Treatment in carbon monoxide (CO) at 350 °C after the calcination step is an experimental trick, once adopted also by industry, to stoichiometrically reduce the Cr(VI) precursors into Cr(II) species amenable to polymerize ethylene already at room temperature and almost without any induction period, two properties that might be useful for producing, for example, ultrahigh molecular weight polyethylene.^48−50,109^ Reduction in CO at 350 °C was widely used in academia because the so-obtained highly coordinatively unsaturated, “naked”, Cr(II) species readily adsorb nitrogen (N_2_), CO, and nitric oxide (NO), forming a variety of nitrogen, carbonylic, and nitrosylic complexes that constituted an optimum playground for testing the sensitivity of several spectroscopic methods.^36,51,59,60,110^ Pioneered by Zecchina in the late 1970s exactly on this system,^111−115^ IR spectroscopy of adsorbed probe molecules is nowadays a well-established technique, which allows surface sites to be distinguished as a function of their coordination environment, with a sensitivity that is probably unsurpassed by any other experimental technique.^36,116,117^ In the following, we will try to summarize the main information which can be retrieved by *in situ* IR spectroscopy of probe molecules applied on the CO-reduced Phillips catalyst and to put them in relation with the concept of active site strain developed in the previous section.

Among the investigated probe molecules, carbon monoxide is by far the most used and probably also that with the best interaction strength, *i.e.*, neither too strong nor too weak, which is important not only to discriminate sites with very similar conformations but also to detect adsorbate-induced structural changes. IR experiments of CO adsorbed on CO-reduced Cr/SiO_2_ samples, hereafter referred to as Cr(II)/SiO_2_, started to appear in the scientific literature in the mid-seventies.^111−113^ At that time, the silica used as a support was mostly Aerosil300, a pyrogenic silica that has nothing to do with those used commercially for the Phillips catalyst but has the advantage to be spectroscopically simpler, in that the very small particle size and absence of porosity determine a negligible scattering in the whole Mid-IR region and above. For these reasons, Areosil300 was continuously adopted also in the successive decades as a support for model Cr(II)/SiO_2_ systems, and is still in use nowadays, even though the number of experiments performed on Cr/SiO_2_ prepared with polymer-grade silicas is no longer counted.

When the Cr(II)/SiO_2_ is correctly prepared (*i.e.*, avoiding the formation of Cr_2_O_3_ clusters and assuring the complete removal of eventually adsorbed CO), adsorption of CO at room temperature gives rise to a characteristic “triplet of bands” around 2180 cm^–1^ (*i.e.*, substantially blue-shifted with respect to the ν(CO) value of the molecule in gas phase, 2143 cm^–1^), which is a function of the CO pressure and is diagnostic of each system and of the activation history of the sample.^51,59,60,107,112,118−120^ The frequency positions of these bands indicate that the carbonyls have a “non-classical” nature, which means that the electrostatic effect (originating from the interaction between the electric field generated by the chromium cation and the CO dipole moment) and the electron donation from the 5σ orbital of CO to 3d orbitals of the metal dominate over the back-donation from 3d orbitals of the metal to the 2π* orbitals of CO.^121^ This behavior is not observed for homogeneous chromium carbonyls and indicates that the silica surface provides uncommon opportunities for stabilizing the Cr(II) species.

**Figure 2:**
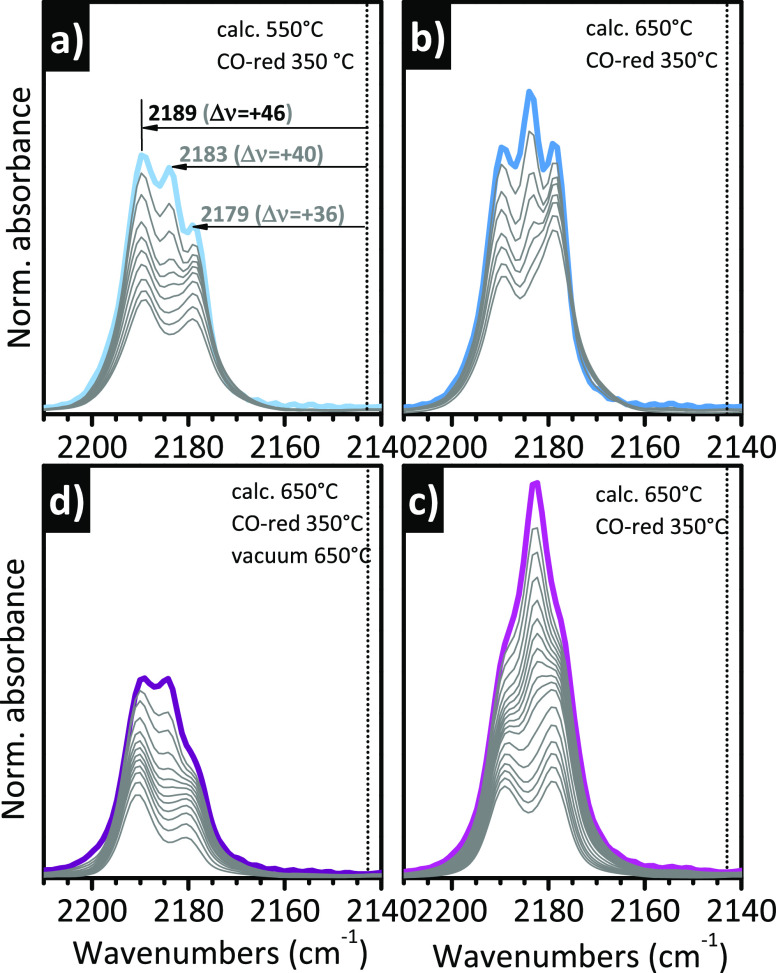
IR spectroscopy allows the discrimination of Cr(II) sites as a function of their coordination environment. The figure shows the IR spectra, in the ν(CO) region, of CO adsorbed at room temperature as a function of the CO coverage on two Cr(II)/SiO_2_ samples subjected to a different thermal history. (a and b) Spectra of a Cr-doped glass monolith (Cr loading of 0.1 wt %) calcined either at (a) 550 or (b) 650 °C and then reduced in CO at 350 °C. Adapted with permission from ref (119). Copyright 2019 Elsevier. The two sequences of spectra have been normalized to the optical thickness of the sample, hence the absolute intensities are comparable. (c and d) Spectra of a Cr/Aerosil300 sample (Cr loading of 1.0 wt %) calcined at 650 °C and (c) reduced in CO at 350 °C or (d) successively treated under vacuum at 650 °C. Data reproduced with permission from ref (59). Copyright 2005 American Chemical Society. The two sequences of spectra have been normalized to the optical thickness of the pellet, hence the absolute intensities are comparable. In all parts, the dotted vertical line indicates ν(CO) of gaseous CO.

Some examples are reported in Figure 2, which shows the characteristic “triplet” for CO adsorbed at room temperature on two distinctly different Cr(II)/SiO_2_ samples, activated following different protocols. All the sequences of IR spectra have been collected upon decreasing the CO coverage.^59,107,119^ These data have been already published, but here they are presented together to highlight the potentials of IR spectroscopy in revealing subtle differences in the population of the Cr(II) sites. The first sample is a Cr-doped glass monolith (obtained by sol–gel method, SSA = 570 m^2^/g) with a chromium loading of 0.1 wt % (*i.e.*, 10 times lower than the most frequently investigated with 1.0 wt % loading)^107,119^ that was calcined at two different temperatures, 550 (Figure 2a) and 650 °C (Figure 2b), in both cases followed by reduction in CO at 350 °C: the comparison serves to illustrate the effect of the calcination temperature on the relative abundance of different Cr(II) species. The second sample is a “standard” Cr/SiO_2_ based on Aerosil300 with a chromium loading of 1.0 wt %, either activated at 650 °C and reduced in CO at 350 °C (Figure 2c) or subjected to a further treatment at 650 °C under vacuum after reduction in CO (Figure 2d):^59^ the comparison is useful to show that the relative abundance of each chromium species can be tailored with postreduction treatments.

In all the cases, three bands are clearly distinguishable at the maximum CO coverage, at 2189, 2183, and 2179 cm^–1^ (*i.e.*, upward shifts of Δν = +46, +40, and +36 cm^–1^, respectively, with respect to gaseous CO), but with different relative intensity from case to case. The three bands have been traditionally assigned to monocarbonyl (band at 2189 cm^–1^) and dicarbonyl species (bands at 2183 and 2179 cm^–1^) formed, respectively, on two types of Cr(II) sites with different conformations, which are named Cr_B_(II) and Cr_A_(II), respectively, in the specialized literature.^59,112^ Upon decreasing the CO pressure, the dicarbonyl species on Cr_A_(II) are converted to monocarbonyl ones (band at 2179 cm^–1^). Only a fraction of the carbonyl species is reversible at room temperature. The inability of Cr_B_(II) to coordinate more than one CO molecule at room temperature indicates either that Cr_B_(II) has less coordination vacancies than Cr_A_(II) or that it is less protruding, the two things not necessarily being correlated. When comparing the spectra obtained on the two different samples activated with the same procedure (Figure 2b and c), two observations can be made. The first is that the three bands are more resolved for the Cr-doped monolith glass, suggesting a higher homogeneity of sites. The second is that the relative intensity of the three bands is slightly different. With the activation procedure being the same, these differences must be attributed to the different support. Indeed, it is expected that the distribution of siloxane rings differs from one silica to the other and surely is different for sol–gel and pyrogenic silicas. This leads to a different distribution of the CR size.

Comparing the IR spectra for the Cr-doped glass monolith activated at two different temperatures (Figure 2a and b), it is immediately evident that the relative intensity of the three bands drastically changes, which indicates that the relative population of the Cr(II) sites is affected by the thermal treatment, as discussed in the sections above. For lower activation temperatures, Cr_B_(II) prevails over Cr_A_(II), while at higher activation temperatures new Cr_A_(II) sites become accessible to CO. A reverse effect is observed when looking at the spectra of CO adsorbed on the Cr(II)/Aerosil300 sample treated under vacuum at high temperature after reduction (Figure 2d). With respect to the triplet observed on the same sample subjected to a standard activation (Figure 2c), a relevant fraction of Cr_A_(II) sites is lost in favor of Cr_B_(II) ones. These results unequivocally reveal that the Cr(II) sites are flexible at the silica surface and can easily rearrange in different (likely less strained) conformations if some thermal energy is provided.

The data summarized in Figure 2 clearly demonstrate that IR spectroscopy of CO adsorbed at room temperature is a very sensitive technique (perhaps the most sensitive one) to experimentally discriminate among Cr(II) sites in different environments. According to the calculation performed by Budnyk et al.,^107^ the monocarbonyl on Cr_B_(II) is well reproduced by an 8CR model, while a 6CR model reproduces better the dicarbonyl on Cr_A_(II) (*vide infra*, Figure 4b). This assignment would be in agreement with the experimentally observed increase of ethylene polymerization activity with increasing the activation temperature: a higher proportion of chromium Cr_A_(II) sites is available. However, it does not explain everything. In particular, the increase in the amount of Cr_A_(II) sites would not explain the fact that lower polymer MWs are obtained with rising the activation temperature; according to the predictions of Huang et al.,^103^ the more strained Cr_A_(II) sites should produce high MW PE. This fact indicates that something is missing in the simplified classification of chromium sites as a function of the CR size.

### 2.4 Adsorbate-Induced Mobility of the Cr(II) Sites and the Role of Siloxane Ligands as Revealed by IR Spectroscopy

**Figure 3:**
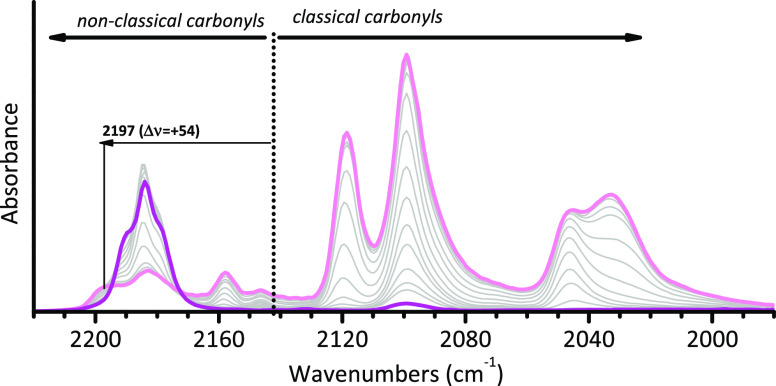
IR spectroscopy reveals the CO-induced mobility of the Cr(II) sites at the silica surface, at the expense of the weaker siloxane ligands. The figure shows the IR spectra, in the ν(CO) region, of CO adsorbed at 100 K as a function of the CO coverage (light pink corresponds to maximum coverage) on a Cr(II)/SiO_2_ sample (Cr loading of 1.0 wt %) calcined at 650 °C and reduced in CO at 350 °C. The spectra are reported after subtraction of that collected prior to CO dosing. Data reproduced with permission from ref (59). Copyright 2005 American Chemical Society. The spectroscopic regions characteristic for classical and nonclassical carbonyls are indicated, with reference to the position of ν(CO) for gaseous CO (dotted line).

When IR spectroscopy of adsorbed CO is performed at the liquid nitrogen temperature, the overall scenario spectacularly changes in a way that, to the best of our knowledge, has no analogs in the literature. Figure 3 shows a typical sequence of IR spectra for CO adsorbed at 100 K on a Cr(II)/SiO_2_ sample as a function of the CO coverage. Without entering into too much detail, which can be found in specialized papers,^36,51,59,60^ we limit ourselves to observe that at 100 K the spectrum at the maximum CO coverage is characterized by a series of intense absorption bands red-shifted with respect to the stretching frequency of CO in the gas phase, indicative of the formation of tricarbonyl species on both Cr_A_(II) and Cr_B_(II) sites, having a “classical” nature.^121^ This spectrum gradually turns back to the original “triplet” observed at room temperature when the coverage is decreased, indicating that the tricarbonyl species are easily reversible. Formation of classical carbonyls, which is common for homogeneous chromium complexes, requires that the interaction of CO with the metal center is dominated by π-back-donation from the 3d orbitals of metal to the 2π* antibonding orbital of CO. This may happen when a structural rearrangement occurs. This leads to more exposed chromium sites, where the overlap between the filled 3d orbitals of Cr(II) and the empty 2π* orbital of CO is maximized, explaining the red-shift of the ν(CO) bands and their high intensity.^36,51,59,60,122^ It has been proposed that this structural change occurs at the expenses of weaker siloxane ligands that complete the coordination spheres of the Cr(II) sites. This has an energetic cost, justifying the easy reversibility of tricarbonyl species.

The sequence of IR spectra reported in Figure 3 not only indicates that additional siloxane ligands complete the coordination spheres of both the less (Cr_B_(II)) and more (Cr_A_(II)) strained sites but also reveals the existence of a third type of Cr(II) sites, named Cr_C_(II) in the seminal literature,^59,112^ not able to adsorb CO at room temperature, instead forming monocarbonyl species at liquid nitrogen temperature (band at 2197 cm^–1^, *i.e.*, Δν = +54 cm^–1^ with respect to gaseous CO). Whether they play a role in ethylene polymerization or not is not well-known at present.

**Figure 4:**
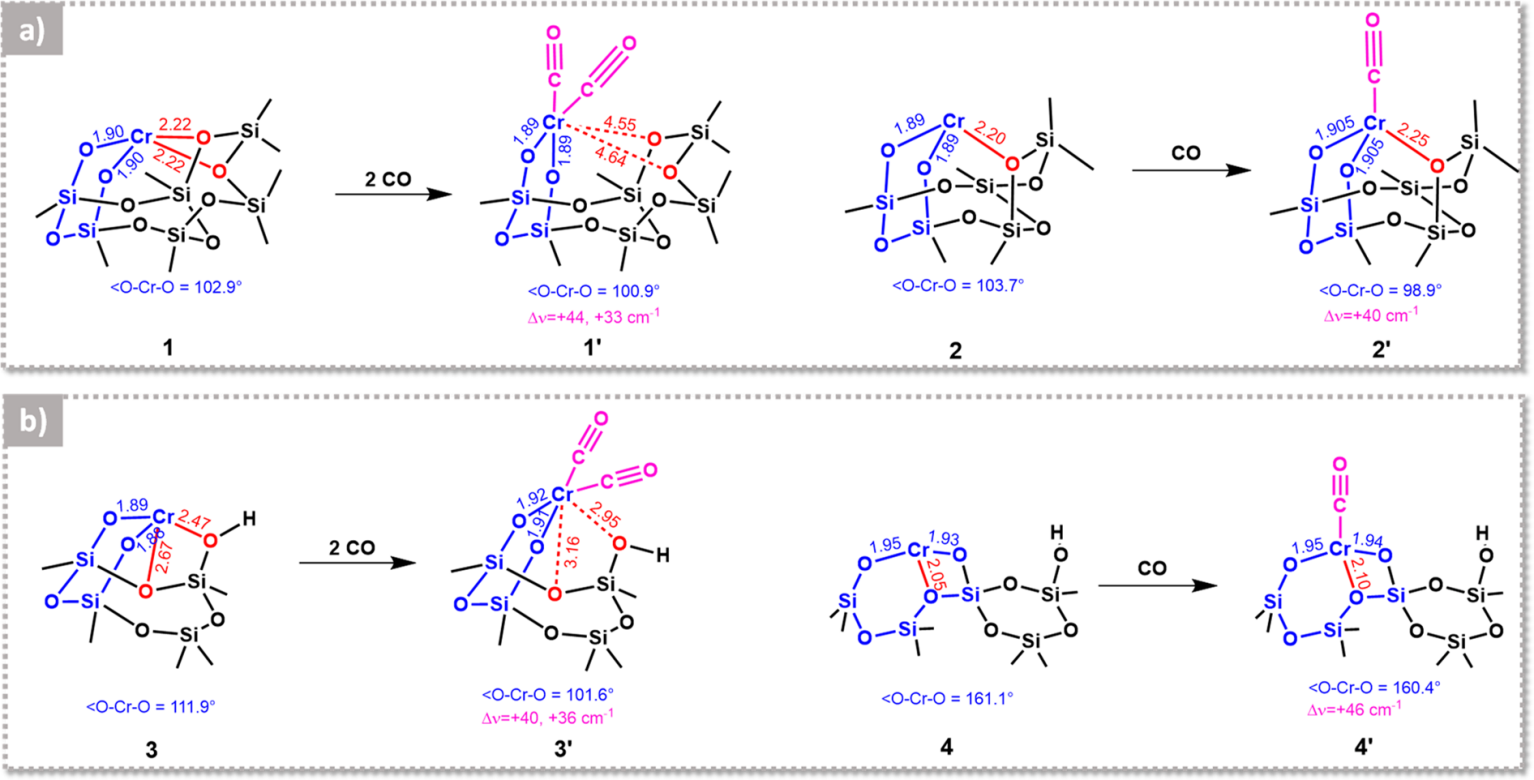
Computational works predict a certain flexibility of the Cr(II) sites at the silica surface. The figure shows several proposed structures for different Cr(II) sites and related carbonyl species as obtained from DFT calculations by (a) Scott and co-workers^120^ and (b) Damin and co-workers^107^. Species **1**–**3** are embedded in a 6CR, while species **4** belongs to an 8CR (in blue). Additional weaker oxygen ligands are shown in red. For species **4**, the Cr(II) is stabilized by an extra oxygen ligand belonging to its own 8CR. Relevant bond distances and angles are reported, as well as the computed Δν(CO) values. (a) Adapted with permission from ref (120). Copyright 2012 Elsevier. (b) Adapted with permission from ref (107). Copyright 2015 Elsevier.

It is important to notice that we have introduced here a new concept not yet discussed in the previous sections, which is the contribution of the weaker siloxane ligands to define the chromium coordination sphere. Siloxane ligands are formed as a consequence of OH condensation during the activation step, and their amount increases upon increasing the activation temperature. Most of the theoretical models discussed in the previous sections neglect the contribution of these ligands and are necessarily simplified, with some exceptions. For the purpose of this Review, we discuss two examples from the literature. To predict the structure of Cr(II) sites on silica, Scott and co-workers^120^ chose two 6CR models coordinated by one or two siloxane ligands, labeled as **1** and **2**, respectively, as shown in Figure 4a. They investigated the adsorption of CO on both models and found that the binding of two CO molecules to model **1** is exothermic by 23.5 kcal/mol and results in the displacement of both siloxane ligands, with a negligible variation in the Cr–O distances. Instead, only one CO molecule binds to model **2**, without displacement of the coordinated siloxane ligand and with a small elongation of the Cr–O distance. These models reproduce the experimentally observed blue-shift of the ν(CO) vibrations, even though they do not perfectly match. In particular, the monocarbonyl **2′** underestimates the experimentally observed Δν(CO) of 6 cm^–1^. Interestingly, the authors notice that the formally less saturated site (**2** in Figure 4a) binds only one CO molecule, while the formally more saturated site (**1** in Figure 4a) binds two CO molecules, suggesting that the number of carbonyls formed in the presence of CO is not necessarily an indication of the formal coordinative saturation of the Cr(II) sites.

Similar results can be found in the already cited work of Damin and co-workers,^107^ who studied the adsorption of CO on a 6CR model, where Cr(II) interacts with a nearby silanol moiety and a siloxane bridge (site **3** in Figure 4b), and on a more buried 8CR model, where Cr(II) strongly interacts with a third oxygen atom belonging to its own 8CR (site **4** in Figure 4b). They found that site **3** easily binds two CO molecules, with a substantial elongation of the Cr–O distance and the displacement of the two weaker ligands, while site **4** binds only one CO molecule, with a negligible variation of the Cr–O distance and without displacing the weaker oxygen ligand. As for the previous two models, also in this case there is no correlation between the formal number of coordination vacancies and the number of bonded CO molecules. However, better than before, these two models are able to reproduce quantitatively both the IR and the UV–vis experimental spectra of CO adsorbed on Cr(II)/SiO_2_, strongly supporting the assignment of Cr_B_(II) sites to less strained CRs than Cr_A_(II) ones.

Taken together, these results add an important piece to the puzzle of the heterogeneity of sites in Cr(II)/SiO_2_ catalysts. Cr(II) sites differ not only due to their strain, which is mainly related to the size of the chromasiloxane ring (which affects the O–Cr–O angle) and influences their activity (Figure 1), but also due to the number of weaker ligands coordinated nearby (either siloxane or OH groups). The size of the CR ring, therefore the strain of the Cr(II) sites, and the number of coordinated siloxane ligands, therefore coordinative saturation, are not necessarily correlated; it seems actually that more buried Cr(II) sites (*i.e.*, larger the O–Cr–O angle) have less need to be stabilized by additional ligands. Moreover, the theoretical calculations summarized above indicate that formation of chromium carbonyls and the displacement of the weaker siloxane ligands are accompanied by an important structural rearrangement of the Cr(II) themselves, and in particular by a narrowing of the O–Cr–O angle and an elongation of the covalent Cr–O bonds that link the Cr(II) sites at the silica surface. This latter phenomenon was quantitatively demonstrated through *in situ* temperature-dependent EXAFS spectroscopy by Gianolio et al. in 2010,^123^ who proved that anchored Cr(II) sites are extracted from the surface upon CO adsorption at room temperature, and even more at 100 K, with an average elongation of the Cr–O bond up to +0.08 Å.

The CO-induced structural rearrangement of the Cr(II) sites is indirectly detectable also by means of vibrational methods. In 2006, Damin et al.^124^ performed a systematic resonant Raman investigation on a Cr(II)/SiO_2_ sample in absence and in the presence of CO. It was demonstrated that two bands in the spectrum of Cr(II)/SiO_2_, at 1009 and 568 cm^–1^, can be ascribed to silica framework modes perturbed by Cr(II) sites. These two bands shift to 1048 and 542 cm^–1^, respectively, upon CO adsorption, revealing that, as expected, the vibrational properties of the anchored Cr(II) sites are strongly perturbed by the presence of an adsorbate.

**Figure 5:**
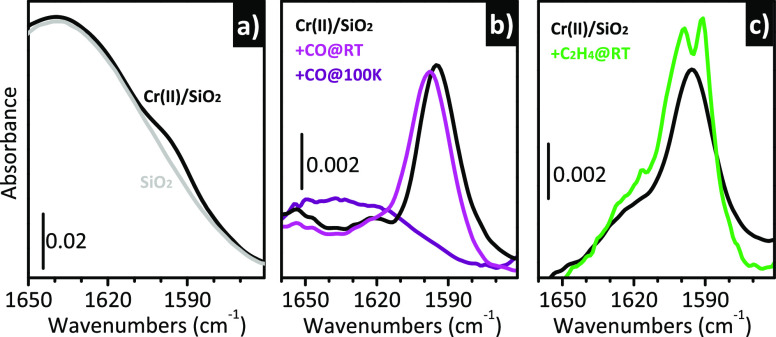
IR spectroscopy indirectly detects the adsorbate-induced mobility of the Cr(II) sites at the silica surface. (a) IR spectra of Cr(II)/SiO_2_ and of the bare silica support in a spectral region dominated by the overtones and combinations of the fundamental (SiO_4_) vibrations of bulk silica. (b) Spectrum of Cr(II)/SiO_2_ after subtraction of that of silica and that of the same sample interacting with CO either at room temperature or at 100 K. (c) The same as (b) for another Cr(II)/SiO_2_ sample, before and after a short interaction with ethylene at room temperature. Unpublished data.

More recently, Piovano and Groppo^122^ pointed out that a similar phenomenon can be detected also by means of IR spectroscopy, even though it is less evident due to the inability to exploit any resonance effect. Figure 5a shows the IR spectrum of Cr(II)/SiO_2_, magnified in a spectral region dominated by the overtones and combinations of the fundamental (SiO_4_) vibrations of bulk silica. The spectrum of the SiO_2_ support activated under the same conditions is also shown for comparison, while the difference between the two spectra is reported in Figure 5b. A weak but sharp band at 1596 cm^–1^ is present in the IR spectrum of Cr(II)/SiO_2_ and not in that of the bare silica. Although very weak, this band is reproducible, as shown by the spectrum in Figure 5c, which belongs to a different experiment. Its assignment is still uncertain; however, two relevant observations can be done: (1) the band is almost absent on samples activated at a lower temperature and/or treated at high temperature after the reduction step and (2) it is responsive to the presence of adsorbates. For these reasons, it can be assigned to a vibration involving protruding Cr(II) sites. Figure 5b also reports the effect of CO at room temperature and at liquid nitrogen temperature: the band shifts upward and decreases in intensity upon an increase in the CO coverage, very much like what happens to the 1048 cm^–1^ band in the Raman spectra. More importantly, the same phenomenon is observed in the presence of ethylene (Figure 5c). In this case, beside the blue-shift of the band at 1596 cm^–1^, a new sharp band is detected at 1591 cm^–1^, which is related to the υ(C=C) stretching vibration of ethylene coordinated to the Cr(II) sites.^110^ This observation indicates that adsorption of ethylene induces structural rearrangements at the Cr(II) sites, “detaching” them from the silica surface and changing their formal coordination state; this act precedes the initiation of the ethylene polymerization, and should be taken into account in any calculation devoted to investigate the mechanisms of self-activation of Cr(II) in the presence of ethylene.

Recently Scott and co-workers^105^ recognized the role played by siloxane ligands in lowering the energy barrier involved in the initiation mechanism and formulated the concept of hemilabile siloxane ligands: siloxanes should be “coordinated just strongly enough to be hemilabile, allowing access to both lower and higher coordination states”. Moreover, Taniike and co-workers^100^ found that the MW of the produced PE is significantly sensitive not only to the presence of a siloxane ligand in the chromium coordination sphere but also to its position. Coordination of the additional oxygen atom at the equatorial position reduces the ethylene coordination energy, therefore increasing the barrier of ethylene insertion and leading to a lower MW. On the other hand, coordination of the siloxane group at an axial position significantly inhibits the chain transfer, leading to a higher MW.

### 2.5 Not Only CO Is Able to Extract the Cr(II) Sites out of the Silica Surface: Mixed Complexes, “Comonomer Effect”, and Role of Oxygenated Ligands Explained by IR Spectroscopy

**Figure 6:**
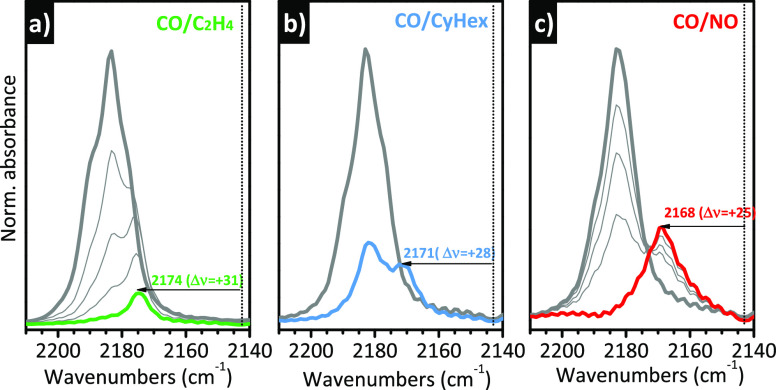
IR spectroscopy reveals that the adsorbate-induced structural rearrangements of the Cr(II) sites at the silica surface is a function of the adsorbate. The figure shows the evolution of the IR spectra of Cr(II)/SiO_2_ interacting with CO at room temperature (bold gray spectra) after contact with a second, stronger, ligand: (a) ethylene, (b) cyclohexene, and (c) NO. All the spectra are reported after subtraction of that collected prior interaction with any probe and in the ν(CO) region. (a) Reproduced with permission from ref (59). Copyright 2005 American Chemical Society. (b) Reproduced with permission from ref (127). Copyright 2016 American Chemical Society. (c) Reproduced with permission from ref (128). Copyright 2010 John Wiley and Sons.

Formation of mixed complexes involving CO and another ligand is a valid method to demonstrate that the adsorbate-induced structural rearrangement of the Cr(II) sites is not limited to the case of CO but has a more general validity. Figure 6 shows three notable examples. Figure 6a represents the evolution of the standard “CO triplet” upon the interaction of the Cr(II)/SiO_2_ + CO system with an overpressure of ethylene. Even though it is known that CO acts as a poison in ethylene polymerization, ethylene is a stronger ligand and has the potential to displace it. This explains the rapid decrease in intensity of the “CO triplet” in the presence of ethylene. Interestingly, this is accompanied by the appearance of a new band at 2174 cm^–1^, which has been ascribed to mixed CO/C_2_H_4_ complexes on a fraction of Cr(II) sites.^59,110,113,125,126^ A similar phenomenon is observed in the presence of cyclohexene (Figure 6b)^127^ and NO^128^ (Figure 6c). Both of them are able to partially displace CO, forming mixed complexes with distinctive ν(CO) bands at 2171 and 2168 cm^–1^, respectively. The ν(CO) values for the three mixed complexes are progressively closer to the ν(CO) of gaseous CO (*i.e.*, Δν = +31, +28, and +25 cm^–1^, respectively). Most probably, ethylene, cyclohexene, and NO cause a structural rearrangement of the Cr(II) sites involving the lengthening of the Cr–O distance and the narrowing of the O–Cr–O angle, a condition that favors the overlap between the filled 3d orbitals of Cr(II) and the empty 2π* orbital of CO, slightly increasing the contribution of π back-donation.

The case of cyclohexene is particularly interesting because it has been claimed by Barzan et al.^127^ to be at the origin of the so-called “comonomer effect”, *i.e.*, the enhancement of the ethylene polymerization rate in the presence of α-olefin comonomers.^129^ The IR spectra reported in Figure 6b suggest that cyclohexene (chosen by Barzan et al.^127^ as a “false comonomer” because it mimics α-olefins without incorporating into the polymer chain) induces a structural rearrangement at the Cr(II) larger than ethylene. According to this vision, α-olefin comonomers “extract” the Cr(II) sites out of the silica surface, increasing their strain and thus enhancing the ethylene polymerization rate.

**Figure 7:**
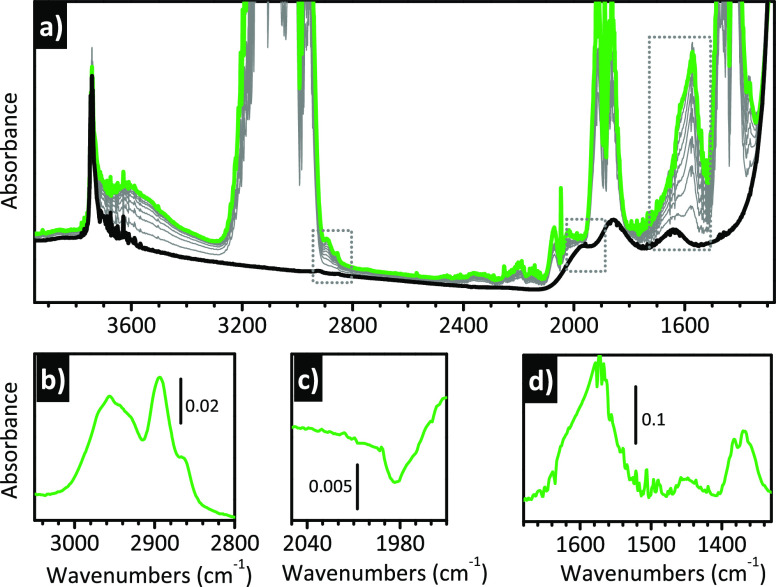
*Operando* IR spectroscopy allows the detection of the formation of oxygenated byproducts during the reduction of Cr(VI)/SiO_2_ by ethylene. These byproducts are responsible for a structural rearrangement of the reduced chromium sites, which precedes ethylene polymerization. (a) *Operando* IR spectra collected during reaction of ethylene with Cr(VI)/SiO_2_ at 150 °C. Color code: black, spectrum collected prior ethylene dosage; gray, spectra collected in the presence of ethylene as a function of time; and green, spectra collected after 30 min. (b–d) Final spectra after subtraction of those collected prior to ethylene dosing, magnified in (b and d) two spectral regions characteristic of the oxygenated byproducts and (c) in the region containing the first overtone of the ν(Cr=O) vibrational mode. Spectra reproduced with permission from ref (130). Copyright 2017 American Chemical Society.

These last examples (ethylene and cyclohexene) show that what could appear as a pure curiosity, *i.e.*, the adsorbate-induced structural transformation of Cr(II) sites, actually has important implications in the field of catalysis. If the catalytic performance of a chromium site depends on its strain and on the number and position of hemilabile siloxane (and/or silanol) groups, it is clear that any external ligand able to “extract” the chromium site from the silica surface, without irreversibly poisoning it, might play a fundamental role. According to Barzan et al.,^130^ a similar role is played by the byproducts of chromate reduction, when the Cr(VI) sites are self-alkylated in the presence of ethylene. Formation of oxygenated species different from formaldehyde (which was the byproduct hypothesized in the early literature) during the induction period was inferred by McDaniel and co-workers by indirect TG, DSC, and MS experiments.^131,132^ Barzan et al.,^130^ applying a multitechnique approach comprising *operando* IR spectroscopy, introduced for the first time the important concept that these oxygenated (and flexible) byproducts remain in the coordination sphere of the reduced chromium sites also during the ethylene polymerization and participate on it. Figure 7a shows the *operando* IR spectra collected during the reduction of Cr(VI)/SiO_2_ by ethylene at 150 °C before the onset of the polymerization. The spectra are dominated by the bands associated with gaseous ethylene (due to the long optical path of the DRIFT cell); however, two intense bands are clearly visible at 1617 and 1573 cm^–1^, and their assignment to oxygenated species is straightforward. These two bands, together with many others of weaker intensity, become more visible upon removing the contribution of gaseous ethylene, as shown in Figure 7b–d. Simultaneously, the spectroscopic manifestation of the chromate species disappears, as reported in Figure 7c, which shows the band ascribed to the first overtone of the ν(Cr=O) vibration. The detailed assignments of the new IR absorption bands appearing during the induction time can be found in the original paper.^130^ Here we limit to saying that they are ascribed to vibrations of oxygenated molecules derived from a disproportionation of formaldehyde on the Cr(II) sites, and in particular methylformate.

## 3 Structural Flexibility of Metal Centers in Zeolites

### 3.1 Zeolites: A Single Name, Thousands of Materials

Zeolites are both natural and synthetic microporous inorganic crystalline materials characterized by a regular network of channels and/or cages of molecular dimensions. Their tridimensional structure is obtained by the interconnection of tetrahedral [SiO_4_]^4–^ and [AlO_4_]^5–^ units, which share bridged oxygen atoms. To date, 248 kinds of zeolites, discovered in nature or synthesized artificially, have been approved by the Structure Commission of the International Zeolite Association (IZA-SC) and collected in a database, where they are named by three capital letters (such as MFI, FAU, and CHA) in order to clarify their uniqueness. The silicon to aluminum ratio cannot be smaller than 1:1 (known as the Loewenstein rule), as two Al tetrahedral atoms cannot share one common oxygen atom. The exact position of aluminum ions in the zeolite lattice is to large extent unknown, although in the last few years many studies have shown that both the synthesis procedure and the reagents can have a role in affecting the Al distributions.^133−135^ In parallel, theoretical works also attempted to describe Al(III) siting, evaluating the most thermodynamically stable distribution of Al(III) and applying a statistical approach to the data generated by molecular dynamics, as well as trying to link theoretical calculation and experiments.^136−138^

The trivalent nature of the Al atom leads to a negatively charged framework that needs to be balanced by counterions.^139^ The latter can be either an equivalent amount of extra-framework cations that make neutral the structure or protons that form OH groups with a strong acidic character.^140−142^ The lack of covalent bonds between the extra-framework cations and the zeolite lattice allows the production of a wide variety of materials through conventional aqueous ion exchange, impregnation, or solid state exchange. In all cases, both the topology and the composition of the zeolitic frameworks play a role in the cation exchange processes, conditioning the number of exchanged species. Moreover, the final location of the cations, once the zeolite has been dehydrated, is structure- and composition-dependent, affecting the reactivity properties afterward.^143^ Metal centers can be also isomorphically substituted in the zeolite framework, replacing a small amount of silicon atoms.

Being crystalline and thermally stable, zeolites are more often regarded as rigid materials, while they are characterized by fast bond breaking and formation,^144^ especially in the presence of reactants. Moreover, valence state transformation, phase evolution, and migration of some species can be observed over these materials even at a moderate temperature. The recognition of labile, dynamic, and flexible behaviors expands their potential toward an even broader and more effective use in catalysis. In this context, vibrational spectroscopies (IR and Raman) can provide very useful insights in respect to the species involved in the reactions and their evolution upon interacting with reagents or probe molecules. In the following, we will show some examples of the kind of information that can be retrieved by applying *in situ* IR spectroscopy to zeolites containing heteroatoms.

### 3.2 Adsorbate-Induced Flexibility of Ti(IV) Heteroatoms in TS-1

Besides Al(III), other atoms such as B(III), Ga(III), Ge(III), and others, including even transition metal elements, such as Fe(III) or Ti(IV) ions, can replace a small amount of the central Si(IV) species, providing peculiar properties exploitable for selective adsorption/exchange or for applications in heterogeneous catalysis. The heteroatoms can be included in the synthesis mixture or introduced with postsynthetic treatments, giving rise to quite different materials in respect to the amount, distribution, and stability of the species.^145^

**Figure 8:**
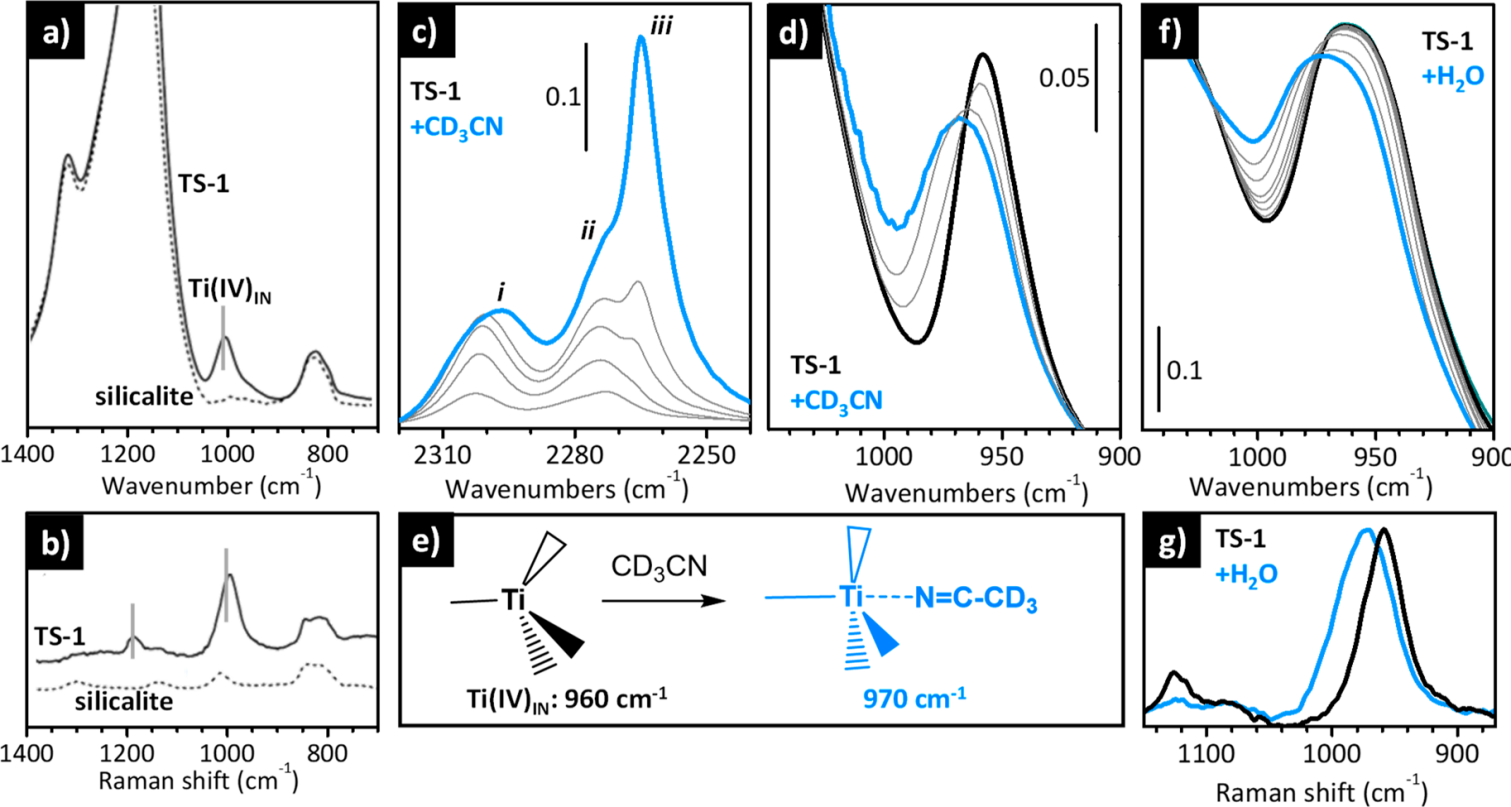
IR and Raman spectra of TS-1 provide information on the local structure of the Ti(IV) sites and on their adsorbate-induced flexibility. (a and b) IR and Raman spectra, respectively, of pure silicalite (dotted) and TS-1 (full) dehydrated in vacuum at 400 °C. Spectra reproduced with permission from ref (146). Copyright 2006 John Wiley and Sons. (c) Background-subtracted IR spectra of TS-1 activated in vacuum at 400 °C upon increasing coverages of CD_3_CN in the ν(C≡N) region (maximum coverage in blue). (d) The same as in (c) in the spectral region characteristic for the zeolite framework modes. Spectra reproduced with permission from ref (147). Copyright 2003 American Chemical Society. (e) Schematic representation of the effect of the adsorption of acetonitrile on the coordination geometry of the Ti(IV) sites in TS-1. (f) Evolution of the IR spectra of TS-1 activated in vacuum at 400 °C upon increasing the coverage of H_2_O (from black to blue). (g) Raman spectra of TS-1 activated in vacuum at 400 °C before (black) and after (blue) interaction with H_2_O. Spectra reproduced with permission from ref (148). Copyright 2002 American Chemical Society.

The insertion of heteroatoms into the lattice perturbs the zeolite framework vibrations in a very peculiar way depending on the nature of the species and their aggregation state. Generally speaking, the framework vibrations of a zeolite contribute in two main regions of their mid-IR spectrum: bands in the 1300–1000 cm^–1^ range are due to the asymmetric stretching vibrations of the [SiO_4_] building block (strong in IR and weak in Raman), while bands in the 850–750 cm^–1^ region are ascribed to the symmetric stretching modes (weak in IR and strong in Raman).^146^ As an example, Figure 8 shows the IR (dotted curve in part a) and Raman (dotted curve in part b) spectra of a silicalite, a pure siliceous zeolite with MFI topology, showing the two spectral regions of interest. When Al is inserted in the framework, no substantial changes are observed in this region (not shown), with the vibrational modes involving the [SiO_4_] units being indistinguishable from those of the [AlO_4_] units. The situation is totally different when a small number of heteroatoms with a significant difference in weight are inserted into the zeolite lattice.

The IR spectrum of TS-1 (full line in Figure 8a), a pure silicalite sample containing a few percentage in weight of Ti, is characterized by an additional band at 960 cm^–1^ (labeled as Ti(IV)_IN_ in Figure 8e) not observed in the spectrum of pure silicalite. This band is ascribed to the asymmetric stretching vibration of the [SiO_4_] units perturbed by the presence of the heavier Ti(IV) sites nearby.^149^ This extra band is observed also in the Raman spectrum (full line in Figure 8b), which shows also a second band at 1125 cm^–1^. This band, which is very sensitive to both the local environment of Ti and the excitation laser, has been associated with the total symmetric vibration of the [TiO_4_] units and it is clearly visible only if the Ti species have a perfect tetrahedral symmetry. Moreover, its intensity is enhanced by one or two orders of magnitude when the Raman spectrum is collected with a laser exciting light (λ_exc_) that falls in the electronic transition of the same species, thanks to the resonance effect. This is the case of the Raman spectrum collected with λ_exc_ = 244 nm, which falls on the tail of an intense band ascribed to the oxygen to titanium charge transfer transition.^149−151^

The isolated Ti(IV)_IN_ sites embedded in the silicalite lattice in a perfect tetrahedral environment do not interact with weak bases such as CO. However, they are able to coordinate stronger bases, such CD_3_CN, H_2_O, and NH_3_. In the case of acetonitrile (Figure 8c and d), the C≡N group interacts directly with the Ti(IV) center, as schematically shown in Figure 8e. This disrupts the tetrahedral symmetry and decreases the perturbative effect of Ti(IV) on the [SiO_4_] units. IR spectroscopy nicely reveals this phenomenon, as displayed in Figure 8c and d. Upon dosing increasing amount of acetonitrile on a TS-1 activated in vacuum at 400 °C, a new band appears at 2302 cm^–1^ (band *i* in Figure 8c), which is ascribed to the ν(C≡N) vibration of CD_3_CN coordinated to the Ti(IV) species. This band is upward shifted by 26 cm^–1^ with respect to that of CD_3_CN interacting with the external silanol groups (band *ii* in Figure 8c) and by 37 cm^–1^ with respect to that of liquid-like CD_3_CN (band *iii* in Figure 8c). Simultaneously, the IR absorption band originally at 960 cm^–1^, which is the fingerprint of perfectly tetrahedral Ti(IV)_IN_ sites, shifts to a higher frequency (Figure 8d), *i.e.*, closer to the band of unperturbed [SiO_4_] units.^147^

An even larger impact on the vibrational properties of TS-1 is observed upon the interaction of Ti(IV) with H_2_O or NH_3_. In these cases the Ti(IV) sites are completely solvated and bond breaking may also occur. The net result is that the Ti(IV) sites assume a pseudo-octahedral geometry, as demonstrated, *e.g.*, by UV–vis and XANES spectroscopies.^148^ The expansion of the Ti(IV) coordination sphere affects the vibrational properties of the material. As an example, Figure 8f shows the evolution of the IR spectrum of TS-1 activated at 400 °C after interactions with increasing amounts of water. Similar to that observed in the case of acetonitrile, the band at 960 cm^–1^ blue shifts to 973 cm^–1^. The same effect is observed in the Raman spectrum (Figure 8g), where in addition the band at 1125 cm^–1^ is also almost completely consumed because the Ti(IV) sites lose the tetrahedral configuration.^148^

These results, complemented by other techniques (especially X-ray absorption and UV–vis spectroscopies) and DFT calculations,^152,153^ demonstrate that the isolated Ti(IV) sites in TS-1 are able to expand their coordination sphere in the presence of sufficiently basic molecules. The distortions of the local structure around the Ti(IV) sites are always compensated by rearrangements of the silicalite framework, which in turn is reflected in the vibrational spectrum. Even though an unanimous agreement has not been reached yet, this phenomenon is considered the key to understand the unique ability of TS-1 to perform extremely selective partial oxidation reactions in mild conditions. When in contact with hydrogen peroxide/water (H_2_O_2_/H_2_O) solutions, isolated Ti(IV) sites form peroxo/hydroperoxo species,^154,155^ expanding their coordination sphere very similarly to what has been discussed for acetonitrile and water,^151,156^ and these sites are considered the active species in selective oxidation reactions.

### 3.3 IR Spectroscopy of Adsorbed Nitrogen Oxides Permits the Speciation of Fe Sites in Fe-Doped Zeolites and Tracing of Their Adsorbate- and/or Thermal-Induced Mobility

**Figure 9:**
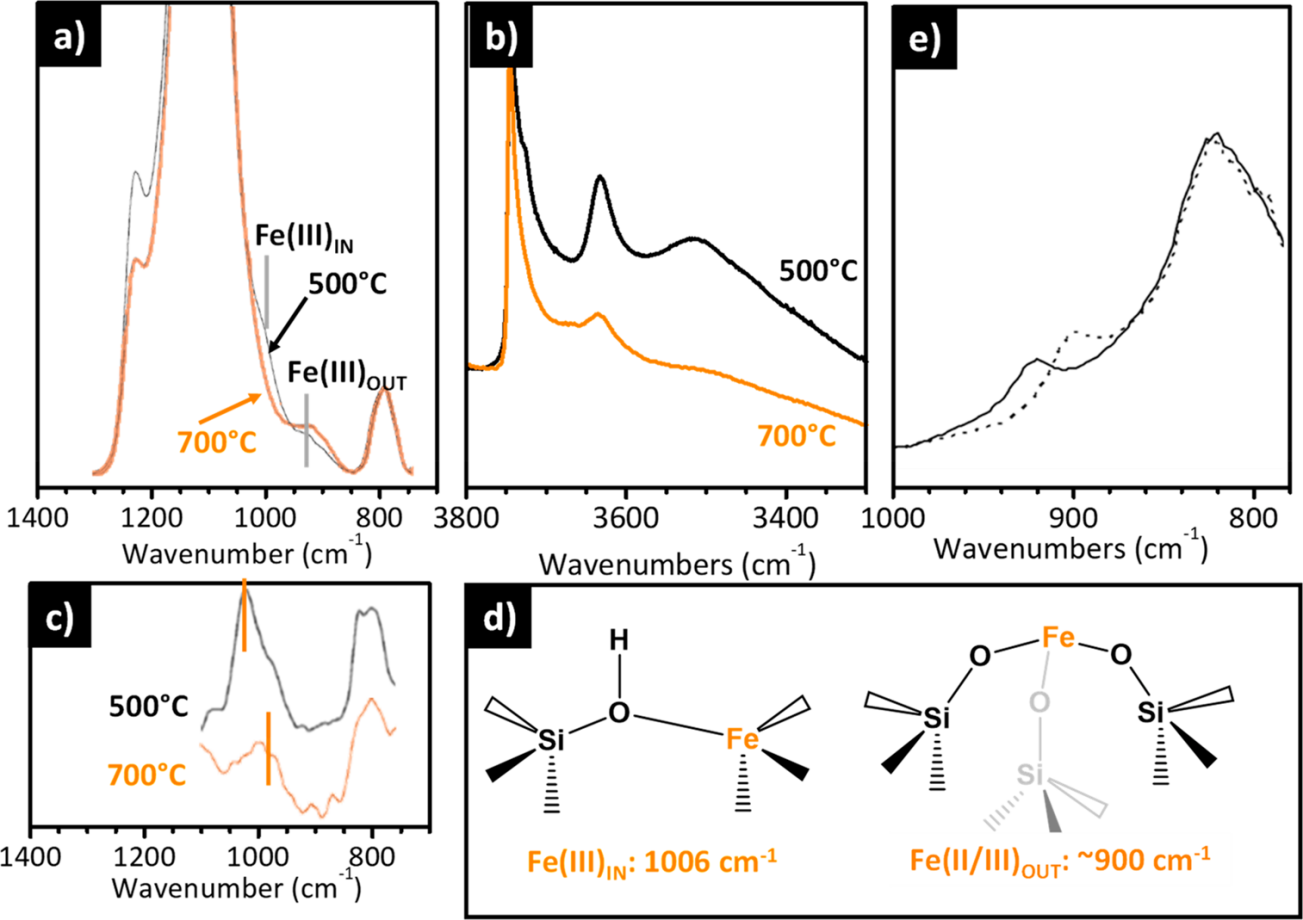
IR and Raman spectra of Fe-substituted zeolites permit the speciation of the Fe sites and tracing of their mobility. (a and b) IR and (c) Raman (λ_exc_ = 1064 nm) spectra of a Fe-silicalite dehydrated in vacuum at 400 °C after calcination at 500 or 700 °C. Reproduced with permission from ref (157). Copyright 1996 Elsevier. (d) Schematic representation of the main Fe species present in Fe-silicalite, depending on the activation conditions, and corresponding IR bands. Fe(III)_IN_ and Fe(III)_OUT_ refer to Fe(III) sites inside and outside the framework, respectively. (e) IR spectra of a Fe/ZSM-5 prepared by CVD before (full line) and after (dotted line) interaction with NO_2_. Spectra reproduced with permission from ref (161). Copyright 2008 Elsevier.

Similarly to Al(III), the insertion of Fe(III) species in the silicalite lattice leads to a negatively charged framework, which generates a certain Bro̷nsted acidity. However, differently from Al(III), which does not perturb the vibrational modes involving the [SiO_4_] units, Fe(III) species affect the framework vibrations, although in a less evident way than Ti(IV). Figure 9a–c show the IR and Raman spectra of Fe–silicalites calcined at 500 and 700 °C. Calcination at high temperature leads to the removal of the template, which is accompanied by a change of the local symmetry of the Fe(III) sites, from almost tetrahedral to a distorted structure with a proton in the close vicinity (Fe(III)_IN_ in Figure 9d). This structural transformation is elegantly demonstrated by XANES spectroscopy, which shows a decrease of the intensity of the pre-edge peak characteristic for perfectly tetrahedral sites.^157,158^ The vibrational fingerprints of the Fe(III)_IN_ species are (i) a band at 3630 cm^–1^ associated with the Si(OH)Fe Bro̷nsted acid sites (Figure 9b) and (ii) bands at 1006 cm^–1^ (in IR, Figure 9a) and 1025 cm^–1^ (in Raman, Figure 9c), which are assigned to the vibrations of the [O_3_Si–O]^−^ units surrounding the Fe(III)_IN_ center^157^ having a *C*_3*v*_ symmetry. This band emerges at the low-frequency side of the main band due to the ν_asym_ vibration of the [SiO_4_] units with *T*_d_ symmetry (1250–1000 cm^–1^) at energies higher than those observed in the presence of Ti.^159^ This is counterintuitive on the basis of the respective atomic mass but can be explained considering the higher ionicity of the bond between Fe(III) species in the lattice and the surrounding oxygen atoms. This implies an intrinsic low frequency of the [FeO_4_] units (IR absorption at 857 cm^–1^) that limits the mixing of ν(Si–O) and ν(Fe–O) vibrational modes.^160^

At lower frequencies, the broad and complex absorption at ∼920 cm^–1^ is ascribed to the lattice vibrational modes of [SiO_4_] units in close vicinity to monatomic Fe(II/III) species not inserted in the framework,^161^ hereafter labeled as Fe(II/III)_OUT_ species. This assignment is supported by the behavior of the IR and Raman spectra after calcination at 700 °C. The band associated with Fe(III)_IN_ species is eroded in favor of that ascribed to Fe(II/III)_OUT_ species (Figure 9a and c), suggesting that calcination at a high temperature promotes the migration of the Fe(III) species from lattice to extra-lattice positions (Figure 9d), in either the II or III oxidation state. Simultaneously, the band at 3630 cm^–1^ associated with Si(OH)Fe Bro̷nsted acid sites decreases in intensity (Figure 9b), which reflects the grafting of extra-framework Fe(II/III)_out_ species to the silicalite surface with consumption of the Bro̷nsted acid sites. Interestingly, a band in the same position (∼920 cm^–1^) is typically observed also in Fe-doped zeolites obtained upon postsynthetic impregnation followed by reductive solid-state ion exchange, or by chemical vapor deposition (CVD), as reported in Figure 9e (full line), which indicates that this is another method to achieve highly dispersed iron cations grafted at the zeolite surface.^161^

The results summarized above demonstrate that IR spectroscopy delivers precious information on the location of the iron cations in Fe-doped zeolites, either in the framework or outside it and grafted at the surface, as well as on their mobility as a function of the treatment conditions. It is worth noticing that similar conclusions on the dynamic evolution of the iron species from the synthesis to the post-synthetic treatments have been achieved by Raman studies, exploiting different exciting lasers able to enhance the intensities of some vibrations thanks to the resonance effect.^150,162,163^

Opposite to what observed for Ti(IV)_IN_ in TS-1, the Fe(III)_IN_ sites inside the silicalite framework are not accessible, because adsorbates mostly interact with the Bro̷nsted site nearby. As a consequence, the IR absorption band at 1006 cm^–1^, which is the fingerprint of Fe(III)_IN_ sites, is not affected by the presence of adsorbates. The situation is markedly different for the Fe(II/III)_OUT_ species grafted at the silicalite surface, which are characterized by empty coordination vacancies. The presence of adsorbates in the coordination sphere of the Fe(II/III)_OUT_ species affects their vibrational properties. As an example, Figure 9e shows the effect of NO_2_ adsorption over a Fe/ZSM-5 material prepared by CVD. The band at ∼920 cm^–1^, ascribed to the lattice vibrational modes of the [SiO_4_] units in close vicinity to monatomic Fe(II/III)_OUT_ species, red shifts to 900 cm^–1^.^161,164^

The accessibility of Fe(II/III)_OUT_ species explains why the interest in Fe-doped zeolites as catalysts has been always higher for materials treated above 500 °C or for materials where iron is inserted by postsynthetic treatments, in which the Fe sites are not placed in the zeolite framework but present either as monomeric Fe(II/III)_OUT_ species grafted at the surface or, eventually, as oligomeric FeO_*x*_ nanoparticles. Seminal works by Panov et al. showed that highly dispersed iron in Fe/silicalite or in Fe/ZSM-5 are able to directly convert benzene in phenol using nitrous oxide (N_2_O) as an oxidant.^165^ More recently iron containing zeolites have been studied for the NO_*x*_ abatement^166^ through selective NH_3_ reduction and for the direct conversion of methane into methanol.^167−169^ The catalytic properties of these Fe-zeolites greatly differ depending on the zeolite topology, composition (*e.g.*, amount of Fe, copresence of Al, or copresence of Cu), and type of pretreatment.

In this context, a standard approach to characterize the Fe species and to compare different materials is the use of IR spectroscopy of adsorbed NO.^170−172^ NO is chosen as a probe molecule because of its strong affinity toward Fe and because the ν(NO) absorption bands are usually intense, which guarantees a high sensitivity also for very diluted samples. Moreover, the greater perturbation of the NO dipole moment when it is adsorbed on Fe(II) rather than on Fe(III) ions makes this approach particularly suited to monitoring the relative distribution of Fe(II) and Fe(III) species.^173,174^ It was demonstrated that partially uncoordinated Fe(II) sites are able to adsorb up to three NO molecules, giving rise to the formation of mono-, di- and trinitrosyls adducts; on the contrary, Fe(III) cations are usually more coordinated and form exclusively mononitrosyl adducts. Finally, as already discussed in the previous section, NO does not interact with the Fe(III)_IN_ species inserted in the zeolite network as substitutional species.

**Figure 10:**
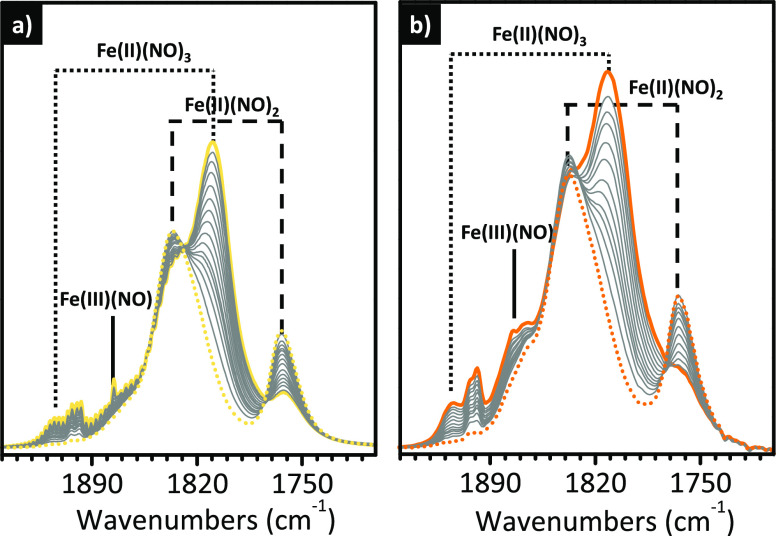
IR spectroscopy using NO as a probe allows the speciation of Fe sites in a Fe-silicalite as a function of the treatment conditions. (a) IR spectra of NO adsorbed at room temperature as a function of the NO coverage (maximum coverage: full yellow; minimum coverage: dotted yellow) for a Fe-silicalite activated at 500 °C. (b) As in (a) for the same material activated at 700 °C. Spectra reproduced with permission from ref (173). Copyright 2002 Elsevier.

Figure 10 shows the potential of IR spectroscopy with NO in the characterization of an Fe–silicalite material. The IR spectra of NO dosed on Fe–silicalite activated in a vacuum at 500 °C (Figure 10a) is characterized by multiple bands undergoing a peculiar evolution upon the progressive decrease of the NO coverage. At the maximum coverage, the spectrum is dominated by a doublet of bands at 1926 and 1810 cm^–1^ (full yellow line), which have been assigned to tricarbonyl adducts of the type Fe(II)(NO)_3_. These two bands decrease in intensity as the NO coverage diminishes, and simultaneously two new bands gradually grow at 1838 and 1765 cm^–1^, which have been ascribed to a dinitrosyl adduct of the type Fe(II)(NO)_2_. The presence of three isosbestic points at 1848, 1834, and 1778 cm^–1^ confirms that dicarbonyl adducts are formed at the expenses of the tricarbonyl ones. Finally, a broad band is also observed in the 1890–1860 cm^–1^ range, which is ascribed to mononitrosyl adducts formed on Fe(III) sites, Fe(III)(NO). When the experiment is repeated on the same material treated with N_2_O (a selective oxidant) at 250 °C before the adsorption of NO, the overall intensity of the spectra drastically decreases, and in particular the absorption bands ascribed to multinitrosyl adducts formed at the Fe(II) sites.^173^ This spectral evolution can be explained by considering that N_2_O selectively oxidizes a major fraction of the Fe(II) sites (which form the Fe=O ferryl sites), which are no longer accessible to NO.

Finally, Figure 10b shows a similar sequence of IR spectra for NO adsorbed on the same Fe-silicalite material activated in reducing conditions at higher temperature (700 °C). The overall intensity of the spectra increases, in particular the two bands ascribed to Fe(II)(NO)_2_ adducts. This spectral evolution indicates that activation at 700 °C causes an increase of the fraction of extra-framework Fe(II/III)_OUT_ species, in perfect agreement with what was discussed above (Figure 9). In some cases, IR spectroscopy of adsorbed NO was adopted in combination with the use of CO, as a probe molecule, in order to identify the distribution of iron species in the zeolitic matrices and their role in catalysis.^175^

These results demonstrate the potential of IR spectroscopy of probe molecules to discriminate between Fe sites in zeolites characterized by a different oxidation state and local environment, similarly to what already discussed in the previous chapter for the Phillips catalyst. More important, this approach puts in evidence that Fe species in Fe-doped zeolites are mobile and can migrate out of the framework, depending on the treatment conditions.

### 3.4 IR Spectroscopy Reveals the Mobility of B Heteroatoms in B-Doped Zeolites

B(III) is another heteroatom that can substitute Si(IV) in a zeolite framework. Boron silicalite (BS-1) has been found to be a promising catalyst for the oxidative dehydrogenation of propane. The active sites are considered to be isolated boron species, which enable the simultaneous activation of molecular oxygen and a carbon–hydrogen bond.^176^

Being lighter than silicon, the presence of boron should perturb the vibrational spectrum of the zeolite in the opposite way than Ti(IV) and Fe(III), *i.e.*, it should cause the appearance of a band at higher energy than that characteristic of the ν_asym_ mode of the [SiO_4_] tetrahedra. However, literature data available on SiO_2_–B_2_O_3_ glasses containing [BO_4_] units contradicts this hypothesis, since the ν_asym_ and ν_sym_ vibrational modes of [SiO_4_] units perturbed by the presence of boron appear at lower frequency than those of standard silicates. This is explained by taking into account that the [BO_4_] units vibrations are fully mixed with the [SiO_4_] ones.^159,177^

**Figure 11:**
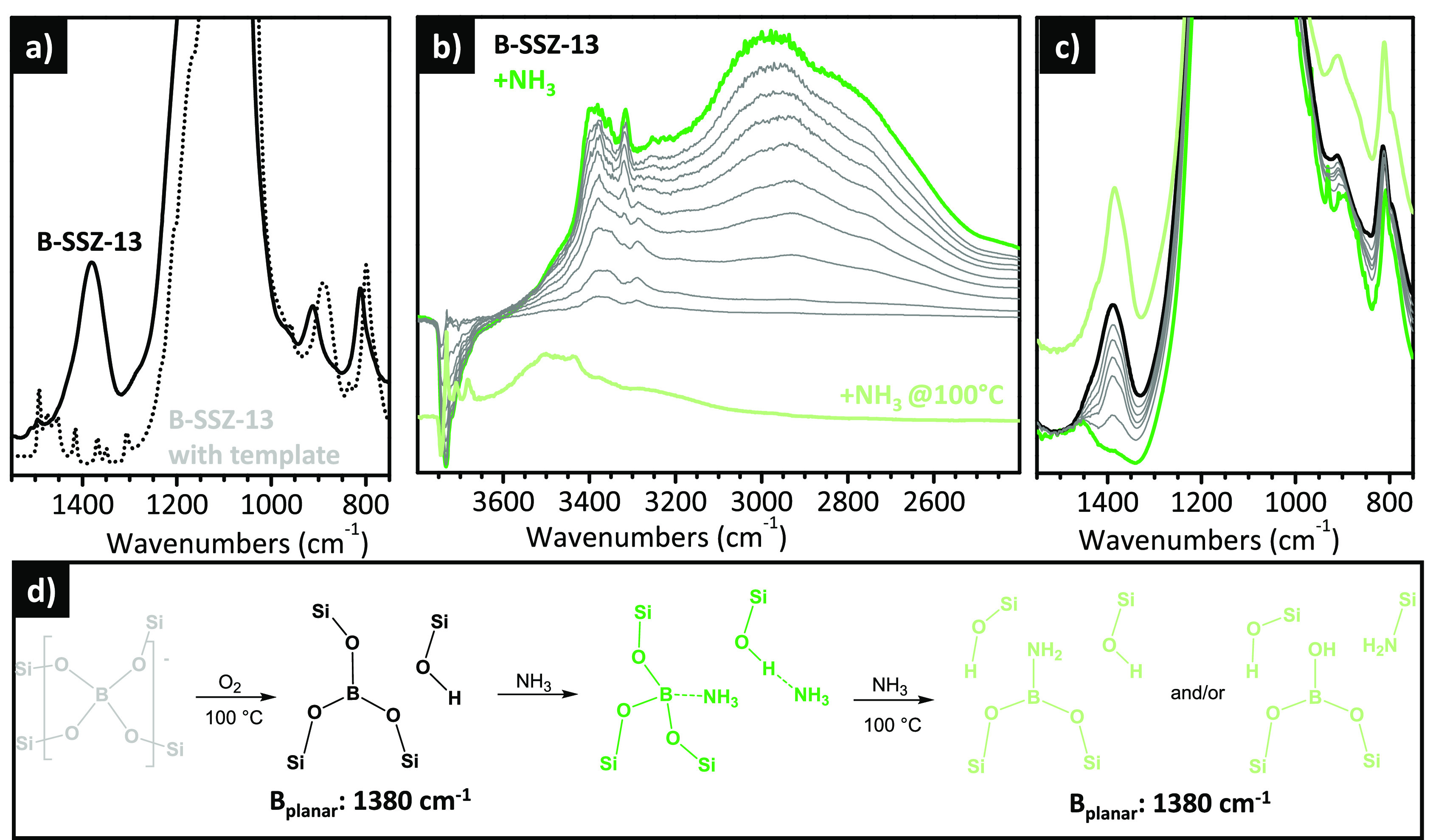
IR spectroscopy reveals the thermal- and adsorbate-induced mobility of boron atoms in B-substituted zeolites. (a) IR spectra of a B-SSZ-13 material as-synthesized (gray) and after calcination at 500 °C (black). Reproduced with permission from ref (178). Copyright 2007 American Chemical Society. (b) Evolution of the background-subtracted IR spectra, in the ν(OH) region, of a B-SSZ-13 activated at 500 °C upon interaction with increasing dosages of NH_3_ at room temperature (full coverage in dark green) and after heating at 100 °C in the presence of NH_3_ followed by degassing (light green, vertically translated for clarity). (c) The same as in (b) in the 1500–800 cm^–1^ region. (d) Schematic representation of the changes in the local structure around the B sites upon template removal, adsorption of NH_3_, and further reaction with NH_3_ at 100 °C. Reproduced with permission from ref (179). Copyright 2007 American Chemical Society.

A similar behavior is observed for B-doped zeolites. Figure 11a shows the IR spectrum of a B-SSZ-13 sample (a zeolite having a CHA topology) still containing the bulky template (gray) and of the same material calcined at 500 °C (*i.e.*, the template was removed, black).^178^ In the spectrum of the as-synthesized sample, the bands ascribed to the ν_asym_ and ν_sym_ modes of the [SiO_4_] units appear systematically at lower frequency with respect to those observed in pure siliceous materials. The same occurs for the band at ∼920 cm^–1^, which is ascribed to the vibrations of the [SiO_4_] units perturbed by silanol groups (observed at ∼900 cm^–1^ for pure siliceous matrices). These three bands upward shift to the values expected for undoped zeolites after calcination and simultaneously a broad band appears around 1380 cm^–1^. These spectral changes have been attributed to an important rearrangement of the boron atoms in the zeolite framework upon template removal, as shown in Figure 11d, from almost perfect [BO_4_] tetrahedral units to [BO_3_] units having a planar geometry (*i.e.*, sp^2^ hybridized B species). The band at 1380 cm^–1^ is assigned to the ν(B–O) vibrational mode of these [BO_3_] units. This structural rearrangement implies the formation of silanol groups with a Bro̷nsted acidity slightly higher than those present at the external surface needed to balance the trivalent boron charge, which confers to these materials a peculiar mild acidity.

The tricoordinated boron sites are accessible by bases, such as H_2_O or NH_3_, and this causes a profound change in the vibrational spectrum. As an example, Figure 11b and c show the evolution of the IR spectra of a B-SSZ-13 activated at 500 °C upon adsorption of increasing dosages of NH_3_.^179^ Beside interacting with the silanol groups through H-bonding (Figure 11b), NH_3_ adsorbs at the B centers, and the band at 1380 cm^–1^ is substantially eroded (Figure 11c). This spectral behavior indicates that, upon the interaction with a medium strong protic base, the tricoordinated planar [BO_3_] species modify their local structure, assuming a trigonally/tetrahedral coordination, as reported in Figure 11d. The absence of the vibrational fingerprint of [NH_4_]^+^ (region not shown) confirms the mild acidic properties of this material. The interaction is partially reversible upon degassing at room temperature.^176^

It has been also demonstrated that a mild thermal treatment at 100 °C in the presence of NH_3_ provides NH_2_-functionalized surfaces on the B-SSZ-13 material, where the NH_2_ groups can be anchored at both B and Si sites, with the simultaneous formation of new Si–OH or B–OH groups, as schematically reported in Figure 11d. This process is associated again with a change in the local structure around the B sites, which recover the initial tricoordinated planar geometry with sp^2^ hybridization. As a consequence, the IR absorption band at 1380 cm^–1^ is recovered (Figure 11c, light green).^179^

### 3.5 Adsorbate-Induced Structural Changes in Cu-Zeolites Tracked by IR Spectroscopy

Cu exchanged zeolites have been extensively studied with respect to two different area of interest in catalysis: NH_3_SCR for removal of nitrogen oxide (DeNOx) emissions of diesel engines^180^ and the direct conversion of CH_4_ into CH_3_OH using O_2_ as an oxidant.^181,182^ Cu metal ions can be introduced into zeolites through solution ion-exchange of solubilized copper salts. This exchange is mostly performed starting from Cu(II) salts, since Cu(I) salts are unstable in air, becoming easily oxidized. After cation exchange and accurate washing, Cu-exchanged zeolites are usually calcined in an oxygen atmosphere to burn any carbon-containing compounds and remove solvents and water. This leaves the bare Cu ions close to the zeolite lattice wall in their oxidized Cu(II) form. Conversely, when the zeolite is heated in an inert atmosphere or under vacuum at high temperature, an autoreduction process is claimed, *i.e.*, the metal is reduced concurrent with the production of O_2_ and/or water.^183−185^ The final aggregation state of the Cu ions and their local environment (first and second coordination sphere) strongly depend on the zeolite topology and the Si/Al ratio.^133,186^ Since all these features have an impact on the catalytic activity and selectivity,^187^ extensive efforts have been devoted to characterize carefully these materials, combining a large variety of spectroscopic methods.^188−190^

**Figure 12:**
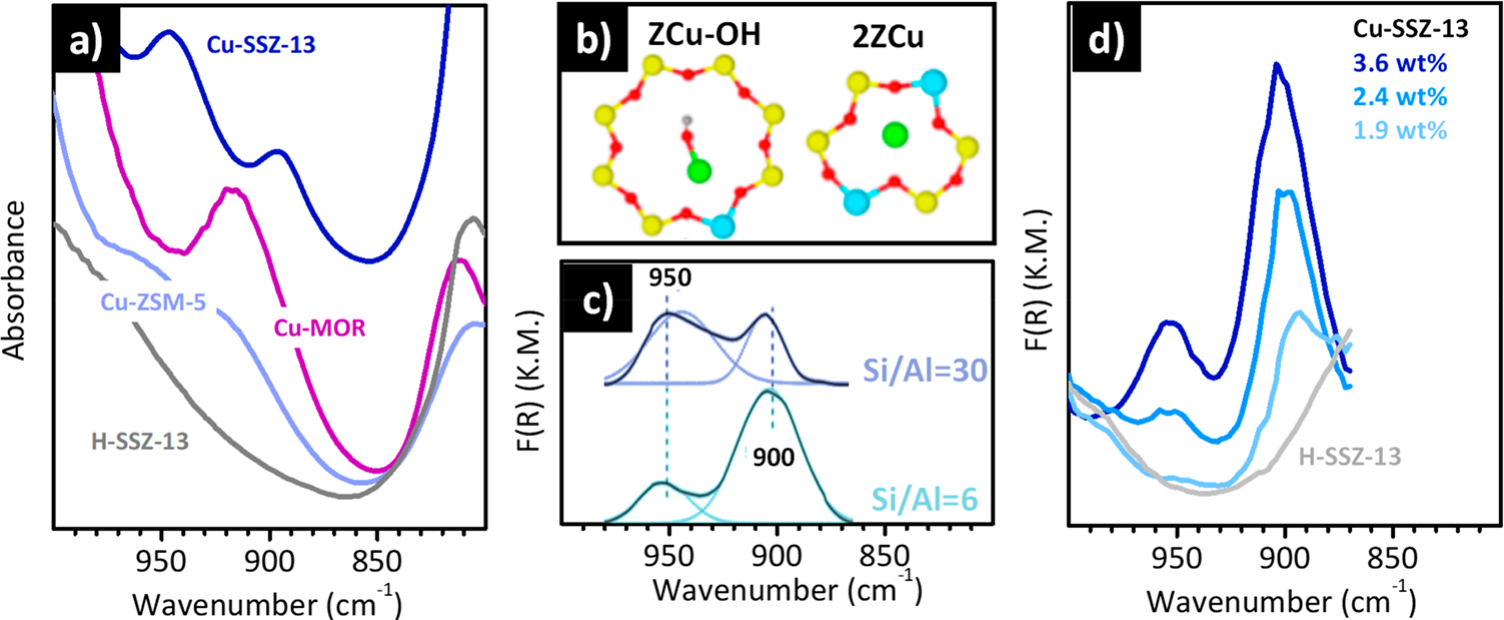
IR spectroscopy allows the speciation and quantification of different Cu sites in Cu-exchanged zeolites. (a) IR spectra, in the [SiO_4_] vibration region, of different Cu-zeolites treated in O_2_ flow at 400 °C. (b) Schematic representation of the two different locations of Cu ions in Cu-SSZ-13 materials (Si, yellow; O, red; Al, light blue; and Cu, green). (c) DRIFT spectra of two Cu-SSZ-13 characterized by different Si/Al ratios. (d) DRIFTS spectra, in the [SiO_4_] vibration region, of Cu-SSZ-13 materials with the same Si/Al = 6 ratio and with various Cu loadings. All the samples have been pretreated in an O_2_ flow at 500 °C. The spectra in (a) are unpublished, while data reported in (b–d) are reproduced with permission from ref (192). Copyright 2018 American Chemical Society.

Among the different approaches, the study of the IR spectra between 1000 and 900 cm^–1^ can be quite informative. Figure 12a compares the IR spectra of a series of zeolites with different topologies (*i.e.*, Cu-ZSM-5 (MFI), Cu-MOR (MOR) and Cu-SSZ-13 (CHA)), all activated in O_2_ at 400 °C. The spectrum of protonic H-SSZ-13 (CHA topology) is also reported for comparison. The spectra of the three Cu-zeolites display one or two bands in the “silica window” that, in analogy to what discussed in the previous sections, are assigned to the υ_asym_ vibrations of the [SiO_4_] units perturbed by the Cu ions in close proximity. The number and position of these bands strictly depend on the zeolite topology. The spectrum of Cu-SSZ-13 shows two bands at 950 and 900 cm^–1^,^191^ which have been assigned to two types of Cu(II) cations: Cu–OH facing the cage and having a single Al atom nearby, [ZCu–OH], and a bare Cu(II) cation in a six member ring of the hexagonal prisms having two Al atoms in the close vicinity, [2ZCu].^192^ The relative intensity of these two bands depends on the Cu distribution in the zeolite, which is affected by both the Si/Al ratio and the overall Cu content. For example, Figure 12c shows the DRIFT spectra of two Cu-SSZ-13 samples with a significant difference in the Al content (Si/Al ratio = 6 or Si/Al = 30) but a similar Cu/Al ratio (30 or 43, respectively). At higher Al content, the band associated with the 2ZCu sites is much more intense because the probability to find two Al sites in the same zeolite ring is higher. Similarly, Figure 12d compares the DRIFT spectra of a series of Cu-SSZ-13 samples with the same Si/Al ratio of 6 but a different Cu loading. The band at 900 cm^–1^, ascribed to the 2ZCu sites, is the only one observed for the sample with a low copper content, while the second band at 950 cm^–1^, ascribed to the ZCu–OH sites, appears at higher Cu loading. This demonstrates that the Cu cations occupy preferentially the 2ZCu positions.^192^

The IR spectrum of Cu-ZSM-5 (Figure 12a) is characterized by two undefined bands at a frequency very close to that observed for Cu-SSZ-13. The featureless nature of these bands has been explained by a poor interaction of the Cu species with the zeolite surface.^193^ Finally, the spectrum of Cu-MOR shows a single band at 920 cm^–1^, assigned to Cu(II) ions located both in the main channels and in the side pockets. The absence of the band at high frequency is compatible with the fact that no υ(OH) bands ascribable to the ZCu–OH species were observed (not shown).

**Figure 13:**
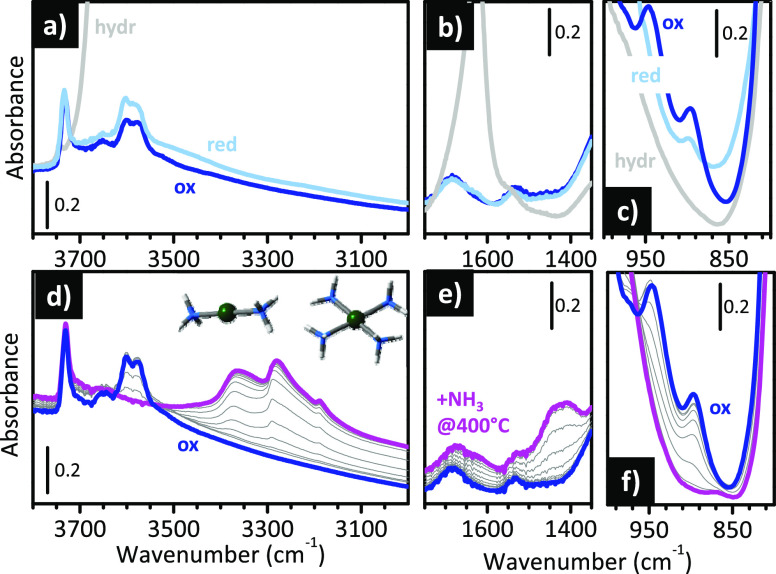
IR spectroscopy is sensitive to the oxidation state and local environment of the Cu sites in Cu-SSZ-13 zeolite. (a–c) IR spectroscopy allows the speciation and quantification of the different Cu sites in Cu-exchanged zeolites. (a–c) IR spectra of Cu-SSZ-13 in its hydrated form (hydr), after thermal treatment in O_2_ at 400 °C (ox), and after prolonged thermal treatment in inert flow at 400 °C (red) in three different spectral regions. (d–f): IR spectrum of Cu-SSZ-13 after thermal treatment in O_2_ at 400 °C (ox), and its time evolution during interaction with NH_3_ at 400 °C. The pink spectrum was collected after 30 min. The insets in (d) show the structure of linear diammino and square-planar tetraammino Cu complexes formed in the presence of NH_3_ at 400 °C. Unpublished data.

The data summarized in Figure 12 demonstrate that the spectroscopic features observed in the 1000–800 cm^–1^ region (also known as “silica window”) are very sensitive to the zeolite topology. In the following, it will be shown that they are equally sensitive to both the Cu oxidation state and the local environment, the latter being determined also by coordinated adsorbates. As far as the effect of the Cu oxidation state is concerned, this is shown in Figure 13a–c, which report the υ(OH) and υ_asym_(SiO_4_) regions of the IR spectra of a Cu-SSZ-13 material in its hydrated form, activated at 400 °C in O_2_ flow or in He flow. In its hydrated form (hydr in Figure 13a–c), the υ(OH) region is dominated by the intense and out-of-scale absorption of physisorbed water, while in the “silica window” region the spectrum appears indistinguishable from that of the protonic forms, as the Cu cations are fully solvated by water and hence do not perturb the vibrational features of the zeolite.

The thermal treatment at 400 °C, either in O_2_ (ox) or in inert flow (red), allows the removal of all the water present in the pores and the formation of well-defined hydroxyl groups, in particular: (i) silanols (band at 3737 cm^–1^), (ii) two types of internal Bro̷nsted sites pointing inside the cage (3611 cm^–1^) or in the hexagonal prism (3584 cm^–1^),^194^ and (iii) Cu(II)–OH species (ZCu–OH), as discussed above (3656 cm^–1^), which balance the extra charge of an isolated Al site in the zeolite lattice.^195^ The relative fraction of these species depends on the type of activation. In the spectral region of the framework vibrations (Figure 13c), the oxidized sample shows the two bands at 950 and 900 cm^–1^ already discussed above. These two bands are much less intense for the reduced sample because the Cu(I) cations have a lower perturbative impact.

More interesting for the purpose of this Review, IR spectroscopy reveals that the interaction between the Cu cations and the zeolitic framework can be modulated by the presence of adsorbates or, in other words, IR spectroscopy is extremely sensitive to adsorbate-induced structural changes involving the Cu cations. This is demonstrated in Figure 13d–f, which show the evolution of the IR spectra of a Cu-SSZ-13 sample, previously treated in oxygen at 400 °C, during its reaction with NH_3_ at 400 °C. This kind of experiment is inspired by the wide use of Cu zeolites in NH_3_-SCR De-NOx catalysis,^196−198^ so that much effort has been devoted to the study of the Cu species formed in the presence of NH_3_ under reaction conditions.^18−20,199^ In the high frequency range, upon increasing the contact time, the bands associated with the strong acid sites and the Cu–OH species are eroded, with the parallel formation of a multiplet of bands associated with υ(NH) of NH_3_ and NH_4_^+^ species. In the δ(NH) region, the growth of an absorption band around 1620 cm^–1^ is ascribable to NH_3_ bonded to Cu(I)/Cu(II) species forming linear diammino or square planar tetraammino complexes that freely float inside the zeolite cavities (inset in Figure 13d),^200^ while the component around 1460 cm^–1^ is due to δ(NH) in NH_4_^+^. It is known that at this temperature we have a mixture of species.^22^ Most likely, all the copper species are involved in the interaction with NH_3_, as demonstrated by the evolution of the spectra in the region of the [SiO_4_] vibrational modes (Figure 13f). Interestingly, the fate of the doublet at 950 and 900 cm^–1^, combined with TPD and molecular modeling calculations, was used to elucidate the nature and the stability of the ZCu–OH and 2ZCu species during SO_2_ poisoning in NH_3_-SCR conditions. It was found that the ZCu–OH species are rapidly poisoned by SO_2_, resulting in the formation of copper bisulfite species that evolve in very stable copper bisulfate in the presence of O_2_. In contrast, the Z2Cu sites react with SO_2_ in the presence of NH_3_.^192^

**Figure 14:**
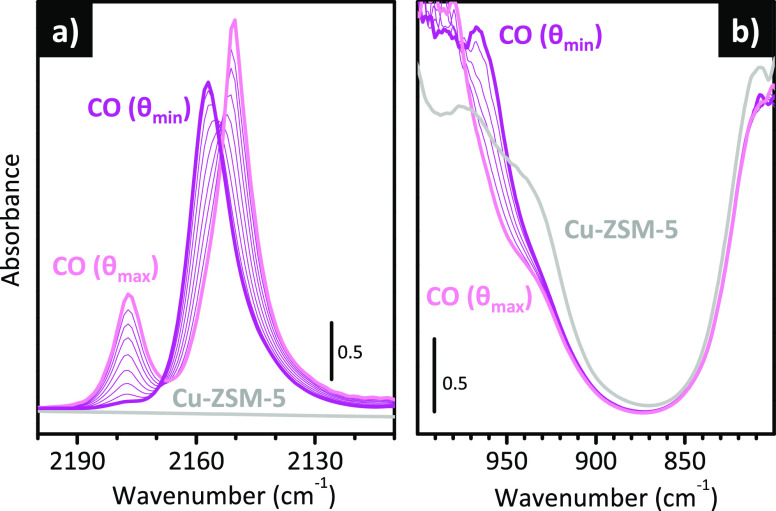
IR spectroscopy reveals the adsorbate-induced mobility of Cu(I) cations in Cu-ZSM-5. (a) IR spectrum in the ν(CO) of a Cu-ZSM-5 preactivated in NH_3_ at 500 °C, that of the same sample in the presence of CO at the maximum coverage, and that after prolonged outgassing at room temperature region. (b) The same as in (a) in the [SiO_4_] vibrational region. Data reproduced with permission from ref (201). Copyright 2018 American Chemical Society.

Finally, IR spectroscopy is also extremely sensitive to the extent of interaction between the Cu cations and the zeolite framework, which is modulated by the presence of adsorbates. This is illustrated in Figure 14, which shows the evolution of the IR spectra of Cu-ZSM-5, previously treated in NH_3_ at 500 °C, in the presence of CO. CO is used here to titrate the amount of Cu(I) species.^201^ At the maximum CO coverage, in the ν(CO) region (Figure 14a), two sharp bands are observed at 2178 and 2151 cm^–1^, which are assigned to the symmetric and antisymmetric stretching of the CO molecules in Cu(I)(CO)_2_ adducts, respectively. Upon decreasing the coverage, the two bands progressively decrease in intensity in favor of a new band at 2157 cm^–1^, ascribed to the ν(CO) of Cu(I)(CO) adducts. The formation of Cu(I) dicarbonyl and monocarbonyl adducts has a profound effect on the vibrational properties of the zeolite framework (Figure 14b). The spectrum of the bare zeolite after the treatment in NH_3_ is characterized by a weak band at 970 cm^–1^, which is ascribed to the [SiO_4_] vibrations perturbed by the presence of Cu(I) cations. Formation of Cu(CO)_2_ adducts leads to an erosion of this band, demonstrating that the Cu(I) cations are brought far away from the zeolite lattice and hence do not perturb it anymore.^202^ Finally, successive removal of the reversible fraction of CO leads to the appearance of a sharp contribution at 967 cm^–1^ that can be associated with the perturbative effect of Cu(CO) on the [SiO_4_] vibrations.

## 4 Ductility of Pt-Based Catalysts in the Presence of (Reactive) Adsorbates

### 4.1 Setting the Scene

Another important class of heterogeneous catalysts that are characterized by a dynamic behavior in the presence of (reactive) adsorbates is that based on supported metal NPs, subnanometric clusters, and single atoms. An increasing amount of postreaction observation and *in situ* or *operando* characterization studies have shown that metal NPs, very much like the single sites discussed in the previous sections, change in the presence of adsorbates and/or under reaction conditions.^2,12−17,203^ Metal NPs are comprise a limited number of atoms and a very large surface-to-volume ratio, which means a high surface energy; hence, they are much more prone to reconstruct than bulk solids. At the single-atom limit, the effect of the reaction environment on the metal state is even more dramatic, with important consequences on the catalytic performances.^11,204,205^ However, while the recent literature is full of successful examples of preparation of single-atom catalysts (SACs), little is known about their stability and the dynamics of isolated atoms under reaction conditions. The number of restructuring possibilities for a metal NP or a SAC in the presence of a reactive environment is enormous: adsorption-induced surface restructuring, bulk atomic restructuring, compound (*e.g.*, oxide, sulfide or carbide) formation, hydrogen absorption, leaching, reactive metal–support interaction, agglomeration, sintering, and redispersion are among the most frequently observed phenomena.^12^

For the purpose of this Review, we will focus exclusively on platinum as the metal and on CO and H_2_ as reactive adsorbates. The metal has been selected because catalysts based on Pt NPs and/or Pt single atoms are prototypical systems in heterogeneous catalysis: Pt is an excellent oxidation as well as hydrogenation catalyst and it is used for numerous applications ranging from car exhaust pollution abatement and petrochemistry to oil refining.^206−209^ As far as the choice of the adsorbates, carbon monoxide is one of the most used probe molecules for characterizing Pt-based catalysts to understand the surface properties and the nature of the active sites. It is also a reactant in many industrial and/or environmental interest processes. In particular, CO oxidation is often employed as a model reaction catalyzed by Pt under mild conditions.^210−213^ On the other hand, Pt-based heterogeneous catalysts are also employed in a wide range of industrial processes involving hydrogen, ensuring high selectivity and conversion at relatively mild operating conditions.^214,215^

Among the possible methods to gain atomic-scale insight into the structure and dynamics of Pt NPs and Pt SACs, aberration-corrected transmission electron microscopy (STEM) has the potential to provide visual evidence of the particle size and morphology. Recent progresses in environmental STEM made it possible to perform measurements under a CO or H_2_ atmosphere, revealing the mobility of the Pt atoms as a function of the gas and temperature. Nevertheless, the method is statistically limited and provides only two-dimensional projections of the objects, from which it is not possible to extract the three-dimensional representation at atomic resolution. Photon-based spectroscopies, such as X-ray absorption (XAS) and X-ray photoelectron spectroscopy (XPS), permit the identification of the average oxidation state and the local coordination of supported metal catalysts. However, these methods have some limitations; for example, they do not allow to differentiate isolated Pt atoms from oxidized Pt clusters, which is often desirable when dealing with catalysts under oxidative reaction conditions. In contrast, IR spectroscopy has the advantage of being a sample-averaged and site-specific characterization technique, which is fundamental to differentiate and quantify the different types of Pt species in a catalyst.^216^ In addition, it can be applied under *in situ* and/or *operando* conditions at a time resolution sufficient to follow the dynamics of the Pt species in most of the reactions of industrial interest.^217^

In the following, we will summarize some of the recent results obtained by applying IR spectroscopy to investigate the ductility of Pt nanoparticles, subnanometric clusters, or SACs in the presence of CO and H_2_. We will start discussing the adsorbate-induced surface and bulk restructuring of Pt NPs, and then we will move to the adsorbate-induced agglomeration of Pt single atoms.

### 4.2 IR Spectroscopy of Adsorbed CO Allows the Differentiation of Adsorption Sites at Platinum Nanoparticles and Subnanometric Clusters

IR spectroscopy using CO as a probe molecule is a powerful and widely used characterization technique that allows the investigation of the local structure, oxidation state, and coordination environment of supported precious metals.^36^ The site-specificity of this method is due to the fact that the vibrational frequency of CO, ν(CO), changes depending on both the adsorption mode and the type of adsorption site, even though it is not always unequivocal.^218,219^ The interaction of CO with platinum extended surfaces has been studied by surface science methods including vibrational techniques for the past 30 years.^220−229^ These studies allowed scientists to define three types of adsorption modes, which are characterized by distinctly different values of ν(CO): (i) top or linear carbonyls (*i.e.*, a single CO molecule is adsorbed to a single Pt atom) usually contribute in the 2100–2000 cm^–1^ region, and (ii) bridge and hollow carbonyls (*i.e.*, a single CO molecule is adsorbed on two or three Pt atoms, respectively), also called multibonded carbonyls, contribute in the 1900–1700 cm^–1^ interval. In all cases we are dealing with classical carbonyls, where the π-back-donation from the d-orbitals of the metal to the 2π* antibonding orbital of CO is largely dominant with respect to σ-donation and electrostatic effects. In addition, a typical band at ∼2120 cm^–1^ (or even higher) has been reported for partially oxidized Pt NP systems and assigned to linear carbonyls on electronical-depleted Pt sites (oxidized Pt^2+^).^230−233^ On regular Pt surfaces, CO molecules adsorb in regular patterns that change depending on the temperature, the partial pressure of CO, and the type of surface. At higher coverage, the top and bridge positions are in competition.^229^ It is worth noting that the IR absorption bands ascribed to multibonded carbonyls are usually much weaker than those associated with linear carbonyls, but this does not imply a smaller amount of the former. In fact, the molar extinction coefficients are different for the two types of carbonyls species.^234^

**Figure 15:**
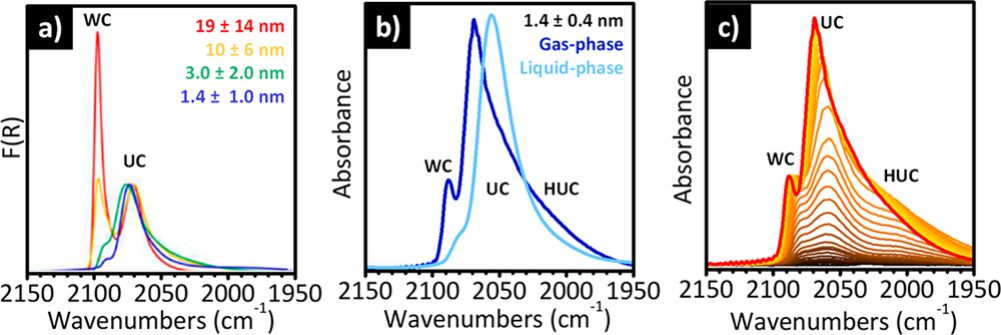
*In situ* IR spectroscopy of adsorbed CO allows the discrimination of adsorption sites at Pt NPs and subnanometric clusters. (a) DRIFT spectra of CO adsorbed at room temperature and at the maximum coverage on four freshly reduced Pt/Al_2_O_3_ samples differing in Pt loading and particle size (see legend). The spectra are normalized to the intensity of the low-frequency band. The high frequency band corresponds to CO molecules adsorbed on well-coordinated (WC) Pt sites, while the band at lower frequency is assigned to CO molecules interacting with under-coordinated (UC) Pt sites. Adapted with permission from ref (237). Copyright 2016 American Chemical Society. (b) IR spectra of CO adsorbed at room temperature and at the saturation coverage on a 5 wt % Pt/Al_2_O_3_ sample with an average particle size of 1.4 ± 0.4 nm, with CO dosed either in the gas-phase or in solution (cyclohexane as solvent). The IR spectrum of CO adsorbed from the gas phase shows an additional band at lower frequency, which is ascribed to highly under-coordinated Pt sites (HUC). (c) Sequence of IR spectra collected upon increasing the CO coverage (from brown to red) on the same 5 wt % Pt/Al_2_O_3_ sample discussed in (b). The data reported in (b and c) are reproduced from ref (242) with permission. Copyright 2022 Royal Society of Chemistry.

Beside differentiating the adsorption mode, IR spectroscopy of adsorbed CO can distinguish the coordination state of Pt surface species.^235,236^ This feature becomes particularly important when dealing with Pt NPs, where the extension of the regular surfaces (with well-coordinated Pt sites) is drastically reduced in favor of defective sites, such as edges and corners, where the Pt atoms are under-coordinated. To illustrate this concept, Figure 15a shows the IR spectra (collected in DRIFT mode) of CO adsorbed at room temperature and saturation coverage on four freshly reduced Pt/Al_2_O_3_ catalysts prepared via incipient-wetness impregnation and characterized by a different average particle size (from 1.4 to 19 nm), achieved by tuning the Pt loading (from 0.05 to 5.0 wt %).^237^ The four spectra are shown in the spectral region characteristic for linear carbonyls. They are all characterized by two main bands, whose relative intensity is a function of the average Pt particle size. The band at high frequency (2100–2080 cm^–1^) is assigned to the collective vibration of CO molecules linearly adsorbed on the so-called well-coordinated (WC) Pt sites (*i.e.*, Pt sites having 8- and 9-fold coordination).^33,238−242^ It is important to notice that, differently than for Pd,^36,243−249^ on Pt it is not possible to distinguish between CO adsorbed at the Pt(111) and Pt(100) surfaces (*i.e.*, the two most favored surfaces) due to their very closed ν(CO) values.^250,251^ Hence, in the following, we will generally refer to CO bound to WC Pt sites without differentiating between CO bound to (100) versus (111) surfaces. The band at lower frequency, instead, is ascribed to CO molecules linearly adsorbed on under-coordinated (UC) Pt sites (*i.e.*, having six- and sevenfold coordination).^238,241,242,252−255^ The lower ν(CO) value is explained in terms of a larger electron transfer from the metal to the antibonding orbital of CO when it is adsorbed on UC Pt sites compared to WC ones.^256^ This in turn increases the static dipole moment associated with the CO vibration and hence the IR extinction coefficient. As a matter of fact, measurements on Pt single-crystals have shown that the IR extinction coefficient is 2.7 times greater for CO adsorbed on edges (UC sites) than on terraces (WC sites).^237,257,258^ From the IR spectra of CO adsorbed at saturation coverage, upon integration of the WC and UC bands and normalization of each area by the proper extinction coefficient, it is possible to quantify the relative fraction of WC or UC Pt sites.^33^ The WC and UC site fractions determined by analyzing the IR spectra reported Figure 15a were in excellent agreement with those predicted by calculation on bare Pt particles of the corresponding size, as obtained from the Wulff construction.^33^

For comparison, Figure 15b shows the transmission IR spectrum of CO adsorbed at room temperature and saturation coverage on a freshly reduced 5 wt % Pt/Al_2_O_3_ sample obtained via deposition–precipitation, with an average particle size of 1.4 ± 0.4 nm (blue spectrum), *i.e.*, comparable to the smallest case in Figure 15a, but with a narrower distribution.^242^ The spectrum is very similar to that reported in Figure 15a for a sample with a similar Pt particle size, except for the presence of a broad tail centered around 2010 cm^–1^ and extending down to 1950 cm^–1^. This band has been ascribed to CO adsorbed at particles corners/kinks,^238,242,255^ hence sites even more under-coordinated (highly under-coordinated, HUC) than those exposed by the Pt particles in Figure 15a. Comparison of Figure 15a and b suggests that both the preparation method (incipient-wetness impregnation vs deposition–precipitation) and the thermal history of the sample (*e.g.*, reduction conditions) affect the types of exposed surface sites in Pt NPs.

The way in which the CO adsorption experiment is conducted is another factor playing a role in determining the position and the relative intensity of the ν(CO) bands. Figure 15b shows the ATR-IR spectrum of CO adsorbed on the same 5 wt % Pt/Al_2_O_3_ sample discussed above, but in the liquid phase instead than in the gas phase, using cyclohexane as a solvent.^242^ Three main differences are observed with respect to the spectrum collected in the gas phase: (1) the two main bands, WC and UC, are downward shifted; (2) the spectrum collected in solution is simpler than that collected in the gas-phase, being the broad band ascribed to CO coordinated to HUC Pt sites almost absent; and (3) the relative intensity of the WC band is much lower than in the spectrum collected in gas-phase. These results indicate that both the WC Pt adsorption sites and the HUC ones are less accessible to CO in the presence of the solvent, which forces CO to adsorb prevalently at the UC Pt sites. In other words, the solvent competes with CO for adsorption on the Pt surface.^259^

Finally, the CO coverage is also a factor influencing the frequency and the intensity of the ν(CO) bands for CO adsorbed onto Pt NPs. This is illustrated in Figure 15c, which shows a series of IR spectra collected upon increasing the CO coverage (θ_CO_) on the same 5 wt % Pt/Al_2_O_3_ sample discussed in Figure 15b. At low θ_CO_, the UC Pt adsorption sites are preferentially populated, followed by population of WC Pt sites at increasing θ_CO_, in agreement with previous reports on supported Pt catalysts^236,241^ and single crystals.^257,258,260^ Moreover, it is evident that, at low θ_CO_, WC and UC bands appear at lower frequencies than at the saturation coverage. The frequency shift observed at high θ_CO_ is due to a strong dipole–dipole coupling interaction between adjacent CO molecules. It is important to observe that this is accompanied by the attenuation of the HUC band and the simultaneous intensification of a band related to UC, which is explained by the “transfer of intensity” concept proposed by Hollins,^261^*i.e.*, a vibrational energy transfer from lower energy to higher energy modes, when dealing with strong dipole–dipole coupling involving more than one adsorbed species.

The discussion above reveals that the nature of the Pt adsorbing sites in catalysts based on supported Pt NPs is well-established in the literature, as well as the use of CO as a probe molecule. However, as anticipated, Pt NPs can undergo restructuring phenomena in the presence of CO under reaction conditions. *In situ* and *operando* IR spectroscopy play a fundamental role also in understanding the transformation between the different species during the different treatments, as will be discussed in the following section.

### 4.3 CO-Induced Restructuring of Pt NPs and Subnanometric Clusters Revealed by IR Spectroscopy

Reconstruction of Pt surfaces due to the adsorption of CO is one of the most studied examples of adsorbate-induced surface reconstruction.^262,263^ The phenomenon was demonstrated to occur on model Pt single crystals using scanning tunneling microscopy (STM), where an increase in the fraction of UC Pt sites was observed for all facets, except (111).^264−268^*In situ* environmental TEM measurements performed at saturation CO coverage have also shown evidence for CO-induced reconstruction on Pt NPs.^269^ It was elucidated that gas adsorption alters surface energies, thus altering the exposed facets.^270,271^ A kinetic barrier of about 0.4–0.5 eV was reported for CO-induced reconstruction of a Pt(100) single crystal,^264^ which suggests that extended Pt surfaces should not reconstruct when CO is adsorbed at room temperature.^33^ However, things may change upon increasing the temperature and/or the CO pressure, as well as decreasing the Pt particle size.

**Figure 16:**
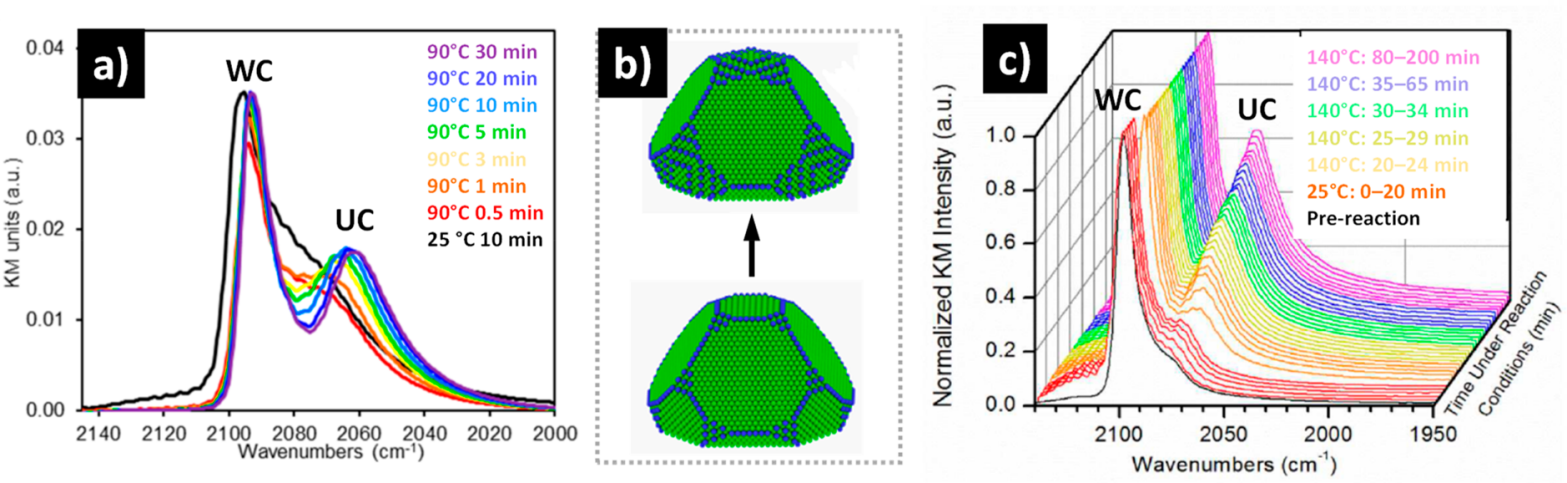
*Operando* IR spectroscopy reveals the occurrence of CO-induced surface reconstruction of Pt NPs as a function of the temperature and under reaction conditions. (a) Evolution of *in situ* DRIFT spectra of CO adsorbed at the saturation coverage on a Pt/Al_2_O_3_ catalyst (average particle size 17 nm) as a function of time and temperature. Adapted with permission from ref (33). Copyright 2017 American Chemical Society. (b) Wulff construction of a 9.2 nm Pt NP based on DFT-calculated free energies for bare surfaces (bottom) and CO-saturated surfaces (top). WC Pt atoms are represented in green, and UC Pt sites are in blue. Adapted with permission from ref (33). Copyright 2017 American Chemical Society. (c) DRIFT spectrum of a Pt/Al_2_O_3_ catalyst (average particle size 19 nm) in the presence of CO at room temperature (saturation coverage, prereaction), and its evolution under CO oxidation reaction conditions (1% CO, 1% O_2_) as a function of time and temperature. Adapted with permission from ref (237). Copyright 2016. American Chemical Society.

*In situ* and *operando* IR measurements may provide an accurate description of the relative distribution of Pt sites under different conditions^272−274^ and, in particular, have been largely employed to study the reconstruction of Pt-based catalysts during the CO oxidation reaction, which is one of the longest established heterogeneous catalytic process.^255,275^ The effect of the temperature on the DRIFT spectra of CO adsorbed on a Pt/Al_2_O_3_ catalyst (average particle size 17 ± 9 nm) while maintaining the CO saturation coverage is shown in Figure 16a. Similar sequences of spectra were reported for samples having a smaller particle size. Upon heating the catalysts to 90 °C in the presence of CO, the absorption band ascribed to carbonyls formed on UC Pt sites becomes more defined and grows in intensity, which was taken as a direct evidence of CO-induced Pt surface reconstruction.^33^ The difference in the fraction of WC and UC Pt sites determined from quantitative IR measurements at room temperature and 90 °C was used by the authors to estimate the amount of reconstruction induced by CO. They found that surface reconstruction is more important for larger particles than for smaller ones (about 10% for 15 nm particles vs 2% for 2 nm particles). The IR results were in excellent agreement with *in situ* STEM measurements and DFT theoretical prediction based on Wulff constructions. Through this correlated approach the authors demonstrated that the truncated octahedron shape adopted by bare Pt NPs undergoes a facet-selective CO-induced reconstruction, which is schematically illustrated in Figure 16b: in the presence of CO the (100) facets roughen into stepped high Miller index facets, while the (111) one remains intact. As a consequence, the amount of UC Pt sites (blue) increases at the expenses of the WC Pt sites (green).

The same authors applied quantitative *in situ* DRIFT spectroscopy to investigate the effect of high CO coverage, under CO oxidation reaction conditions, on the relative concentration of WC and UC Pt sites in Pt/Al_2_O_3_ samples characterized by a different particle size.^237^Figure 16c shows the DRIFT spectrum of a Pt/Al_2_O_3_ catalyst (average particle size of 19 nm) in the presence of the reaction mixture (1% CO, 1% O_2_) at room temperature and CO saturation coverage and its evolution as a function of time when the temperature is increased to 140 °C. Exposure to the reaction mixture at room temperature does not change significantly the IR spectrum. However, as soon as the catalyst is heated up to 140 °C, the band ascribed to CO adsorbed at the UC Pt sites becomes sharper and increases in intensity, indicating that a surface reconstruction is occurring.

Quantitative analysis of the spectra as a function of time indicates that the fraction of WC Pt sites decreases from 89% of the bare Pt NPs to 69% within the first 20 min and then remains stable for the next 3 h of experiment. This indicates that restructuring of the Pt NPs occurs on a very rapid time scale, *i.e.*, well before reaching steady-state conditions. Once again it was found that the largest relative amount of surface reconstruction occurs for the largest Pt NPs (19 nm), where the relative fraction of UC Pt sites increases of about 380% compared to measurements performed before the reaction. Finally, the amount of WC Pt sites evaluated by IR spectroscopy during the catalytic reaction was found to correlate well with the measured TOFs. This demonstrates that the main active sites for the CO oxidation on Pt/Al_2_O_3_ catalysts are the WC Pt sites, which is in agreement with the generally accepted Langmuir–Hinshelwood mechanism of the reaction, requiring the adsorption of CO and its reaction with atomic oxygen. Hence, the efficiency of a catalytic site depends on the CO interaction strength: too strong adsorption sites are poisoned by CO at low temperature and are no longer able to dissociate O_2_. The CO desorption step has the lowest activation energy on WC Pt sites, explaining their higher efficiency in the CO oxidation reaction.

Upon decreasing the Pt NP size, the CO-induced restructuring involves the whole particle and not only its surface. This phenomenon was elegantly discussed by Chizallet and co-workers, who calculated the phase-diagram for alumina-supported subnanometric Pt_13_ clusters in a large temperature and pressure interval. They predicted that already at room temperature and at a relatively low CO partial pressure (much lower than 1 bar), the Pt clusters uplift from the support and exhibit a much higher amount of UC sites compared to the situation found in the absence of CO.^228^ The restructuring of the Pt clusters becomes progressively more relevant upon increasing the temperature and/or the CO relative pressure.

At this stage it is important to remark that the conclusion based on *in situ* IR measurements that CO-induced restructuring of the Pt NPs, increasing the fraction of UC Pt sites at the expenses of the WC ones, is consistent with XAS results on very small (1–3 nm) Pt NPs, where an increase in the Pt–Pt bond distances and in the structural disorder was observed in the presence of CO.^276−278^ This is the opposite of what was found in the case of H_2_, which leads to a decrease of the disorder due to a reconstruction of the Pt NPs into more regular cuboctahedra geometries (*vide infra*).^279,280^ Similarly, first-principle calculations on subnanometric Pt_13_ clusters on γ-Al_2_O_3_ predicted a strong elongation of the Pt–Pt distance with the CO coverage, accompanied by a complex evolution of the Pt–Pt coordination number and an overall increase in the average distance between Pt atoms and the surface of the alumina support. These results suggest the occurrence of a kind of disaggregation of subnanometric Pt clusters in the presence of strongly interacting adsorbates.^228^

### 4.4 IR Spectroscopy of Adsorbed CO Allows the Differentiation of Pt Single Atoms from Pt-Oxide Clusters

IR spectroscopy of adsorbed CO has proven to be particularly suitable for differentiating single metal atoms deposited on supports from oxidized or partially oxidized metal clusters. SAC refers to catalysis by ultradispersed metal atoms, *i.e.*, all atoms are exposed to reactants and available for driving catalytic reactions, hence the metal dispersion is about 100%. It is a relatively recent field of research, fueled by the desire for perfect metal utilization and by the promise of unexpected catalytic performances. The number of experimental reports on Pt SACs has increased exponentially in the last years. A growing number of literature works report on the synthetic strategies, spectroscopic characterization, and reactivity of isolated Pt catalysts for a large number of applications.^281−291^ An inspection to these works indicates that the unambiguous identification of isolated Pt sites is one of the greatest challenges. In particular, distinguishing the IR absorption bands of CO when it is adsorbed on isolated Pt sites, oxidized Pt clusters (hereafter Pt_iso_ and Pt_ox_, adopting the nomenclature of Cristopher and co-workers) or subnanometric Pt clusters is not straightforward. Both Pt_iso_ and Pt_ox_ species contribute in the ∼2080–2130 cm^–1^ interval,^290^ while CO on Pt metal usually contributes in the 2030–2100 cm^–1^ range, with about 20 cm^–1^ of overlap among each others. Nevertheless, most of the works agree on that the ν(CO) bands associated with Pt_iso_, Pt_ox_ and Pt can be discriminated on the basis of two properties: (i) the full-width-at-half-maximum (fwhm) and (ii) their behavior during desorption, which in turn is related to the CO adsorption strength.

**Figure 17:**
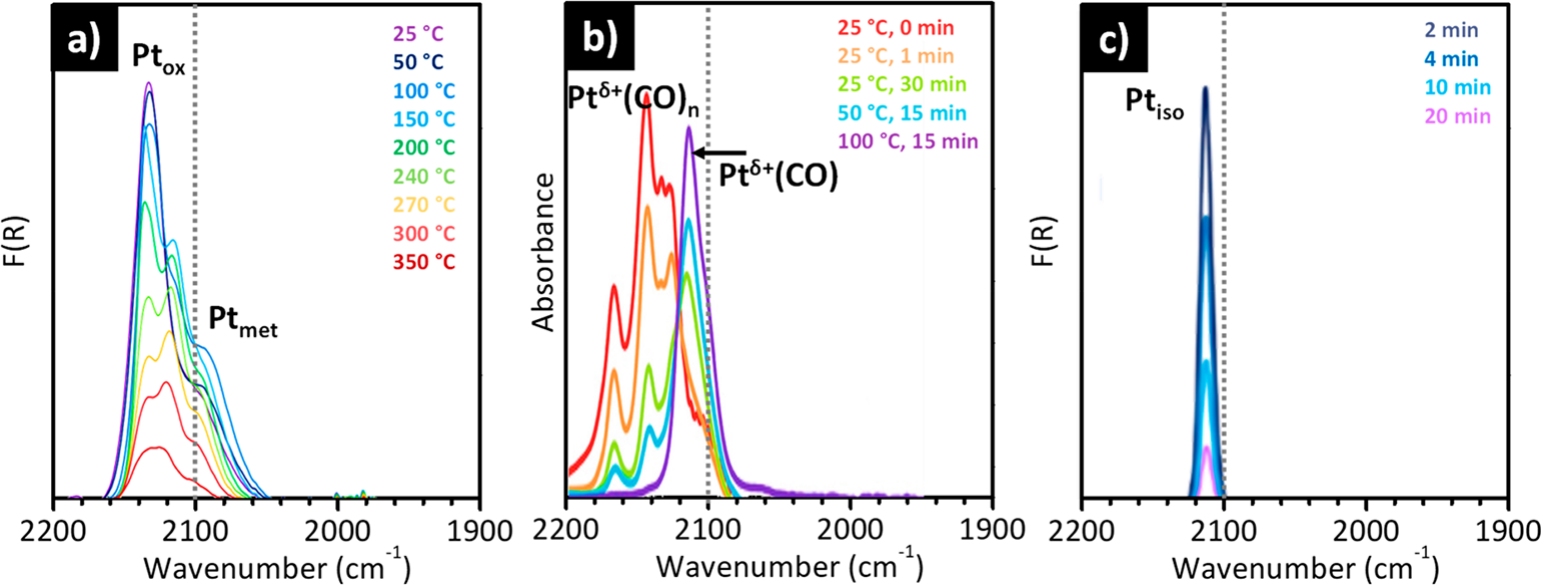
*In situ* IR spectroscopy of adsorbed CO allows the discrimination of isolated Pt sites from oxidized clusters. (a) IR spectra collected at different temperatures during a CO-TPD experiment in He performed on a 1 wt % Pt/TiO_2_ catalyst obtained via incipient wetness impregnation that was preoxidized for 2 h in air at 300 °C. CO desorbs from Pt_ox_ sites at higher temperature than from Pt metal sites, with significant desorption only above 200 °C and incomplete desorption even by 350 °C. Adapted with permission from ref (290). Copyright 2017 American Chemical Society. (b) IR spectra collected at different temperatures during a CO-TPD experiment in Ar performed on a 0.5 wt % Pt/HZSM-5 sample prepared by solution grafting of a Pt organometallic complex. Cationic Pt^δ+^-polycarbonyl species are easily converted into Pt^δ+^-monocarbonyls already at room temperature, the latter being stable at 100 °C. Adapted with permission from ref (207). Copyright 2015 American Association for the Advancement of Science. (c) IR spectrum of CO adsorbed at room temperature on a prereduced 0.05 wt % Pt/TiO_2_ catalyst prepared according to a synthetic protocol, which allows the deposition of less than 1 Pt atom per TiO_2_ particle, and its evolution upon desorption in inert atmosphere at room temperature. Adapted with permission from ref (290). Copyright 2017 American Chemical Society.

A few interesting examples are reported in Figure 17. Figure 17a shows the IR spectra collected at different temperatures during a CO-TPD experiment performed on a 1 wt % Pt/TiO_2_ catalyst obtained via incipient wetness impregnation, which was preoxidized for 2 h in air at 300 °C.^290^ The spectrum of CO adsorbed at room temperature is dominated by a broad band centered at about 2125 cm^–1^, which was associated with CO adsorbed on (partially) oxidized Pt clusters (Pt_ox_), and by weak bands below 2100 cm^–1^, indicative of some residual metallic Pt sites. Upon increasing the temperature under inert flow, the bands associated with CO adsorbed on metal Pt decrease faster than those associated with Pt_ox_. This indicates that CO is bound more strongly on cationic Pt_ox_ sites than on metal Pt. Christopher and co-workers proposed that the strong adsorption energy of CO on Pt_ox_/TiO_2_ is due to the fact that on these partially oxidized clusters, some cationic Pt species are present in under-coordinated geometries.^290^ During the desorption, various shifts in the CO band frequency and changes in the band widths are observed, which suggests evolution in the Pt oxidation states and structure of adsorption sites.

A completely different scenario is obtained when CO is adsorbed at room temperature on a 0.5 wt % Pt/HZSM-5 sample prepared by solution grafting of a Pt organometallic complex,^207^ as shown in Figure 17b. The isolated Al atoms in the zeolite framework provide isolated binding sites for Pt, offering the possibility to obtain Pt_iso_ sites if the Pt loading remains sufficiently low. The spectrum at the saturation coverage displays a series of bands well above 2100 cm^–1^, which are ascribed to nonclassical polycarbonyl species on a variety of Pt cations in different oxidation states.^207,292,293^ Formation of Pt polycarbonyls is a peculiar behavior observed on Pt/zeolites and stems from the concomitance of two factors, the large cationic radius of Pt^δ+^ and their under-coordinated geometry, both of which favor the simultaneous coordination of more than one CO molecule to a single cationic site. These bands gradually decrease in intensity upon desorption in inert atmosphere, and completely vanish as the temperature is increased to 100 °C. Simultaneously, a new band appears at 2115 cm^–1^, which eventually becomes the only one in the spectrum collected at 100 °C. This spectral evolution, which is fully reversible upon re-exposure to CO, has been interpreted in terms of transformation of the cationic Pt^δ+^-polycarbonyl species into Pt^δ+^-monocarbonyls, which are clearly stable until at least 100 °C. It is important to say that the latter band is characterized by a relatively broad fwhm (∼25 cm^–1^), signifying that the Pt_iso_ species occupy a range of different sites on the zeolite support.

Finally, Figure 17c shows the IR spectrum of CO adsorbed at room temperature on a prereduced 0.05 wt % Pt/TiO_2_ catalyst prepared according to a synthetic protocol, which allows the deposition of less than one Pt atom per TiO_2_ particle, and its evolution upon desorption in inert atmosphere at room temperature. At saturation coverage, a single very sharp band (fwhm ∼8 cm^–1^) is observed at 2112 cm^–1^, which was ascribed to Pt_iso_ sites very homogeneous in nature (*i.e.*, located at a common adsorption site on TiO_2_). The band completely vanishes upon flushing He at room temperature for 30 min, clearly indicating that CO binds more weakly on these Pt_iso_ sites than on Pt_ox_ (Figure 17a) or Pt_iso_ hosted inside zeolites (Figure 17b). In addition, throughout the desorption step, neither shifts in frequency nor changes in the shape and fwhm of the band are observed. This is suggestive that CO molecules are spatially isolated on Pt_iso_,^290,292,293^ opposite to what observed for Pt_ox_ sites. The weak adsorption energy between CO and Pt_iso_ sites suggests that these cationic Pt species are strongly coordinated to oxygen atoms in TiO_2_, *i.e.*, their strong coordination to the support reduces the binding energy of CO.

The examples reported in Figure 17 are not exhaustive of the complex scenario present in the specialized literature. In particular, CO adsorbed on Pt_iso_ sites have been reported to contribute at different values depending on the type of support, even at frequencies below 2100 cm^–1^, *i.e.*, at a frequency which is usually associated with CO bounded to metallic Pt. For example, Qiao and co-workers, in a study on a Pt/TiO_2_ catalyst,^294^ attributed the band at 2119 cm^–1^ to CO adsorbed on Pt_ox_ species and the band at 2096 cm^–1^ to CO adsorbed on Pt_iso_ sites. Grunwaldt and co-workers, studying a Pt/CeO_2_ catalyst, assigned a band at 2090 cm^–1^ to CO adsorbed on Pt_iso_ sites,^295^ and Zhang et al. also made this assignment for a band in the same position in the case of a Pt/A_2_O_3_ catalyst.^296^ Interestingly, Karim and co-workers^291^ reported a band at 2082 cm^–1^ (fwhm = 20 cm^–1^) for CO adsorbed on a 0.025% Pt/TiO_2_ sample containing almost exclusively Pt_iso_ sites, in marked contrast with the 2112 cm^–1^ value reported by Christopher et al. (Figure 17c)^290^ for a sample prepared on the same TiO_2_ anatase support. In the same work, they reported signals in very similar positions for CO adsorbed on Pt/TiO_2_ samples characterized by a higher Pt loading, where Pt is present as NPs with an average size of 0.9 nm. Nevertheless, in this case the band also ascribed to CO adsorbed on Pt_iso_ rapidly vanishes upon flushing an inert gas at room temperature, while that attributed to CO adsorbed on Pt clusters does not change in intensity at all under the same conditions. This indicates once more that the binding energy of CO onto Pt_iso_ sites is much weaker than on Pt clusters. The authors ascribe the difference in the position of the ν(CO) band with respect to the work of Christopher as due to the different pH used during the synthesis of the catalyst, which might cause the dissolution of the TiO_2_ support and the creation of different Pt anchoring sites.

*In situ* or *operando* IR spectroscopy has been often used to establish whether Pt_iso_ is more or less reactive than Pt clusters or NPs.^290,297−300^ Once again, one of the applications that received particular attention for the Pt-based catalysts is the CO oxidation reaction, not only because of its simplicity but also because of a practical interest in exploiting Pt SACs in automotive CO oxidation catalysis. Interestingly, the literature reports conflicting results. For example, Stair and co-workers used IR spectroscopy with CO as a probe molecule to analyze a series of Pt-based catalysts supported on different inorganic oxides.^207^ In most of the cases they confirmed the coexistence of both Pt_iso_ and Pt NPs. They also observed that only the CO molecules adsorbed on the Pt NPs are able to react with oxygen to generate CO_2_ under low-temperature conditions, while those adsorbed on Pt_iso_ remain intact. Thus, they concluded that Pt NPs are the active species for CO oxidation, whereas Pt single atoms act as spectators. Similar conclusions were reached by Corma and co-workers for a Pt/Al_2_O_3_ catalyst,^301^ where Pt clusters and NPs presented higher TOFs than Pt_iso_ sites. Similarly, by performing *operando* XAS/DRIFTS on different Pt/TiO_2_ samples, Zhou et al.^302^ concluded that Pt NPs are the active species for the CO oxidation. The same conclusion was reached by Wang and co-workers on different Pt/TiO_2_ samples using *in situ* DRIFTS measurements^303^ and by Grundwaldt and co-workers on Pt/Al_2_O_3_.^304^

**Figure 18:**
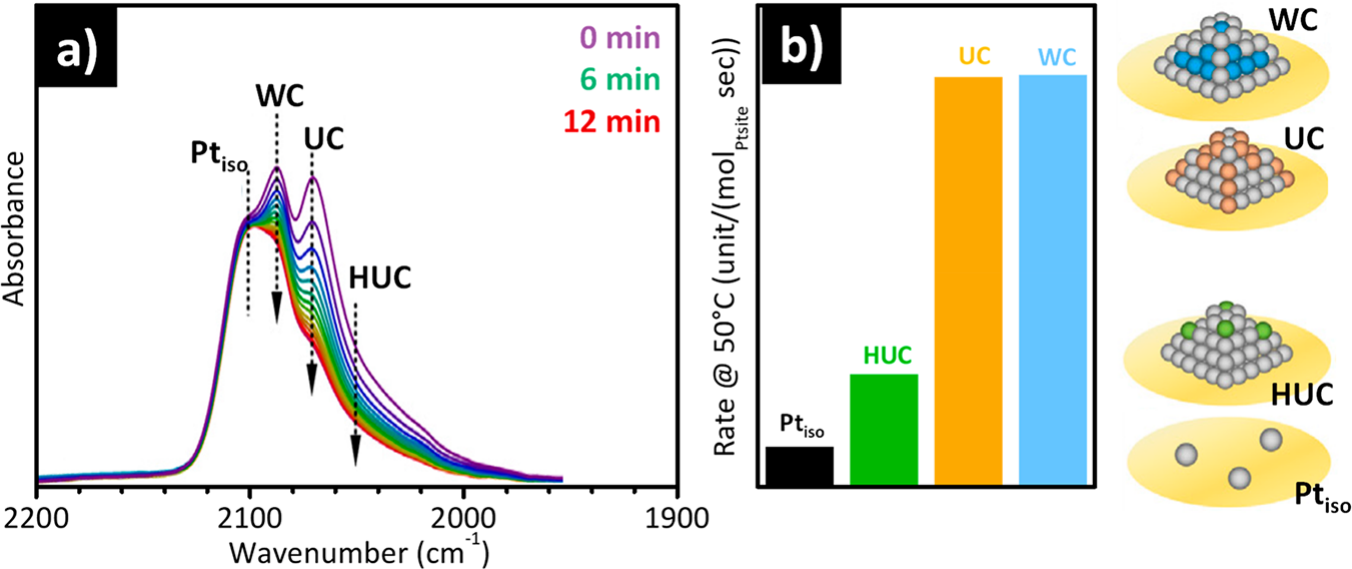
*Operando* IR spectroscopy allows the identification of the Pt active sites in the CO oxidation reaction. (a) *Operando* DRIFT spectra collected during the CO oxidation reaction at 50 °C on a Pt/CeO_2_–Al_2_O_3_ sample. The band at 2100 cm^–1^ is ascribed to CO adsorbed on Pt_iso_ sites, while the bands at lower frequencies are assigned to CO interacting with WC, UC, and HUC Pt sites. (b) Consumption rate of adsorbed CO based on the numbers of different Pt sites in the same Pt/CeO_2_–Al_2_O_3_ catalyst, at 50 °C in the first 5 min after the introduction of O_2_. Adapted with permission from ref (305). Copyright 2022 Elsevier.

Liu and co-workers carried out a series of *in situ* DRIFTS experiments during CO oxidation at low temperature on different Pt/CeO_2_–Al_2_O_3_ catalysts.^305^ The main results are summarized in Figure 18. The IR spectrum of CO adsorbed at 50 °C on the Pt/CeO_2_–Al_2_O_3_ catalyst (violet in Figure 18a) shows IR absorption bands at 2102 cm^–1^, which are attributed to CO adsorbed on Pt_iso_ sites, and bands at 2085, 2069, and 2050 cm^–1^, which are assigned to CO adsorbed on WC, UC and HUC Pt sites in Pt NPs and subnanometric clusters, respectively. As soon as O_2_ is introduced in the reaction mixture, the bands related to CO adsorbed on WC and UC sites on the Pt NPs decline in intensity at the same rate, suggesting that these sites have the same activity for the CO oxidation reaction. In contrast, the band associated with CO interacting with HUC sites declines at a slower rate, and that due to CO adsorbed on single Pt_iso_ sites does not show noticeable change upon O_2_ adsorption. Figure 18b shows the consumption rate of adsorbed CO at 50 °C determined by the IR data. The reactivity of WC and UC sites on Pt NPs is practically the same and much higher than that on HUC Pt sites and Pt_iso_. As a conclusion, the authors claimed that at low reaction temperature Pt_iso_ sites are almost inactive.

The opposite conclusion was achieved by other authors, who claimed that Pt_iso_ species are actually the active species for the CO oxidation reaction.^306,307^ For example, Zhang et al. reported that Pt_iso_ on a FeO_*x*_ support presents a considerably higher TOF value when compared with a Pt/FeO_*x*_ material containing mainly Pt clusters and NPs.^308^ Christopher and co-workers demonstrated that a Pt/TiO_2_ catalyst containing exclusively Pt_iso_ sites is 2 times more active than a similar material containing Pt clusters.^290^ CeO_2_, in view of its special electronic properties, has been also employed as inorganic support to efficiently disperse Pt_iso_ species. According to Nie et al., a Pt/CeO_2_ catalyst containing mainly Pt_iso_ species shows a high activity under low-temperature conditions.^309^ Clearly, reducible metal-oxide supports play an important role in stabilizing Pt_iso_. However, considering that Pt_iso_ on nonreducible inorganic oxides act as mere spectators, reducible supports should also play a role in the catalytic cycle, for example through the generation of active oxygen vacancies.

### 4.5 The Contribution of IR Spectroscopy with CO in Revealing the Aggregation of Pt Single Atoms under Reaction Conditions

**Figure 19:**
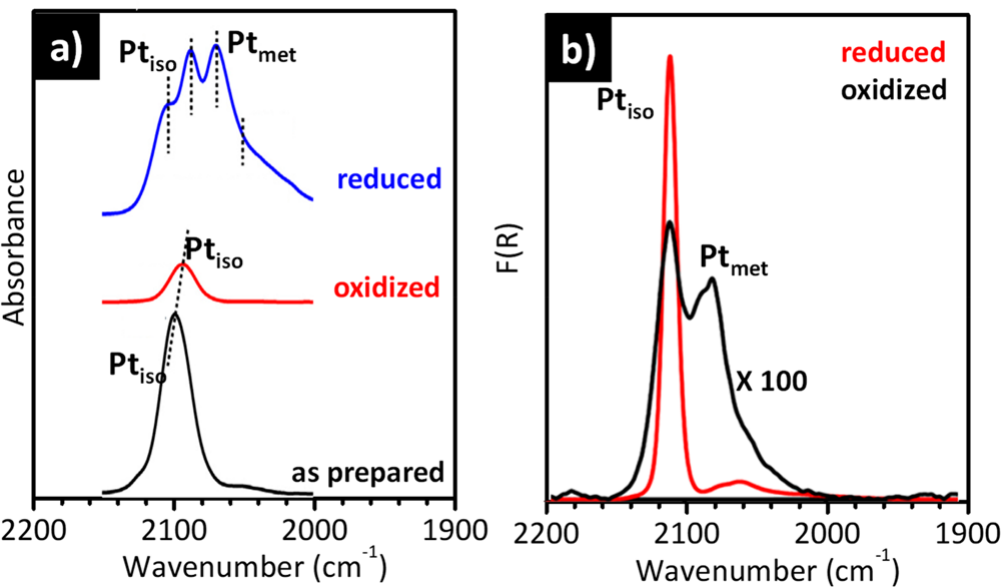
*In situ* IR spectroscopy of adsorbed CO reveals a certain mobility of the Pt_iso_ sites as a function of the activation conditions. (a) *In situ* DRIFTS spectra of CO adsorbed at room temperature on a Pt/CeO_2_–Al_2_O_3_ catalyst as-prepared (black), and on the same catalyst aged at 800 °C for 12 h in air (red) or reduced in 10% H_2_ at 400 °C for 1 h (blue). Adapted with permission from ref (305). Copyright 2022 Elsevier. (b) *In situ* DRIFTS spectra of CO adsorbed at room temperature on a 0.05 wt % Pt/TiO_2_ catalyst that was reduced (red), and oxidized (black), sequentially. The intensity of the latter spectrum is multiplied by 100 to allow comparison. Adapted with permission from ref (290). Copyright 2017 American Chemical Society.

One of the main critical aspects in the development of Pt SACs, which also explains why it is so difficult to rigorously evaluate their reactivity, is their inherent instability and tendency to agglomerate during oxidation (calcination), reduction, or exposure to reaction conditions.^12,301,307^ The adsorbate-induced sintering of isolated Pt atoms is another field of research where *in situ* IR spectroscopy may offer unprecedented insights that are not achievable by means of any other characterization technique. Some examples from the recent literature are reported in Figure 19. Figure 19a shows the *in situ* DRIFT spectrum of CO adsorbed at room temperature on a Pt/CeO_2_–Al_2_O_3_ catalyst (black spectrum): a single IR absorption band is observed at 2097 cm^–1^, which was ascribed to CO adsorbed at Pt_iso_ sites, in accordance with STEM results.^305^ When repeating the same experiment but with the catalyst calcined in air at 800 °C for 12 h (red), a single ν(CO) band is still observed but with a much lower intensity and downward shifted of about 5–6 cm^–1^. This indicates that after calcination the Pt species are still isolated, but they rearranged at the surface, resulting in a considerable increase in electron density of Pt_iso_ with the consequent strengthen of the π-back-donation effect. After reduction in H_2_ at 400 °C for 1 h, the IR spectrum of CO adsorbed at room temperature (blue) drastically changes, showing the characteristic absorption bands ascribed to CO adsorbed on metallic Pt clusters. This example clearly demonstrates that Pt_iso_ sites are mobile also on CeO_2_-related supports, and that the nature of the supported Pt species strongly depends on the reaction conditions. Figure 19b shows the IR spectra of CO adsorbed at room temperature on the same 0.05 wt % Pt/TiO_2_ catalyst discussed in Figure 17c, which was prepared according to a synthetic protocol that allows Pt_iso_ sites to be obtained almost exclusively.^290^ As discussed above, on the freshly reduced sample (red line), a single sharp ν(CO) band is observed at 2112 cm^–1^, which was assigned to CO adsorbed to Pt_iso_ sites. However, when the same sample is oxidized, the overall intensity of the spectrum decreases by 100 times (black line). This behavior was explained by a migration of the Pt_iso_ species in less accessible anchoring sites.^290^

Under certain conditions, CO itself may induce the mobilization and aggregation of previously isolated Pt sites. As demonstrated by *in situ* XAS studies, this is associated with the reduction of Pt cations by CO; reduced Pt species are more mobile than cations and hence prone to agglomeration.^11,301^ Isolated Pt and sub-nanometric Pt species exhibit a higher mobility than Pt NPs under the same conditions. As an example, Corma and co-workers^307^ used *in situ* IR spectroscopy to study the Pt reconstruction in a Pt/Al_2_O_3_ sample under real CO oxidation reaction conditions. The presence of absorption bands at 2120–2150 cm^–1^ in the as-synthesized material are associated with CO interacting with Pt_iso_ sites. Under reaction conditions at 225 °C, instead, ν(CO) absorption bands at 2075 and 2050 cm^–1^ are observed, corresponding to CO adsorbed on WC and UC Pt sites on Pt NPs. This indicates that agglomeration of Pt_iso_ sites occurred. Interestingly, when the temperature is increased to 325 °C, the Pt NPs further undergo a CO-induced surface reconstruction: the fraction of UC Pt sites increases at the expenses of the WC Pt sites, as discussed in previous section. As anticipated above, the stability of isolated Pt atoms can be increased by selecting the right support; in general, reducible metal oxides, such as CeO_2_ or TiO_2_, afford a stronger bonding of the Pt sites, resulting in the preservation of the dispersed Pt atoms.^204,309^ However, despite the greater stability, Pt sintering has been observed also on CeO_2_, or CeO_2_-related supports.^310^

### 4.6 H_2_-Induced Restructuring of Pt NPs Revealed by IR Spectroscopy

CO is not the only adsorbate able to induce a mobility of the Pt species and a restructuring of Pt NPs. In the last decades a large amount of computational and experimental evidence has been accumulated that has demonstrated that Pt clusters on different supports also undergo electronic and morphological reconstruction in the presence of H_2_.^276,280,311−319^ While CO adsorbs more or less strongly on the Pt sites without being activated, molecular H_2_ at room temperature (or above) and subatmospheric pressure undergoes homolytic dissociation at the Pt surface, with the consequent formation of different types of Pt-hydride species, which are those directly involved in hydrogenation reactions.

The first model developed to explain H_2_ adsorption on small Pt NPs was the so-called “three-site model” reported by Koningsberger and co-workers,^320,321^ who, on the basis of XAS data, proposed the existence of three types of Pt-hydrides. Strongly adsorbed linear hydrides (or “atop”) are formed at very low coverage. These species are converted into multicoordinated hydrides at intermediate coverage. Finally, at high coverage, the previously freed “atop” sites are refilled, but this time the Pt–H interaction is much weaker, giving rise to linear hydrides labeled as “on-top”. This model was improved later on by the groups of Raybaud and Sautet,^280^ who systematically investigated by DFT calculations the thermodynamic stability of a Pt_13_/Al_2_O_3_ system as a function of the temperature and of the H_2_ coverage. As anticipated in the sections above, on the dehydrated Al_2_O_3_ surface they found that the most stable configuration for a Pt_13_ cluster is biplanar and strongly anchored to the support. In the presence of H_2_, linear and multicoordinated Pt-hydrides are formed that gradually solvate the Pt_13_ cluster. At high H_2_ coverage, the Pt_13_ cluster dramatically reconstructs to assume a cuboctahedral morphology. Calculation predicts that the reconstruction brings a weakening of the Pt–Pt bond with the consequent elongation of the Pt–Pt distance, as demonstrated experimentally by means of XAS,^278,312,314,322,323^ and also that the Pt_13_ particles are lifted from the support due to the presence of Pt hydrides at the particle-support interface. Similar computational results were obtained by Wang and Johnson^318^ on carbon-supported Pt_37_ nanoparticles.

Even more interestingly, according to calculation, the H_2_-induced morphological reconstruction of the Pt clusters is accompanied by a conversion of top Pt–H species into multicoordinated Pt–H ones. However, the experimental identification of the Pt–H species and their interconversion as a function of the reaction conditions is a challenging task, which requires a site-specific characterization method. As demonstrated in the previous sections, *in situ* IR spectroscopy has the potential to distinguish between different adsorption sites. Nevertheless, in contrast with the huge amount of works exploiting IR spectroscopy coupled with CO adsorption to characterize Pt-based catalysts, the method has been rarely used to directly characterize the Pt–H species,^324−329^ and experimental evidence of H_2_-induced restructuring of Pt-based catalysts were limited for a long time mainly to XAS. Two main factors make the detection of Pt–H species challenging by IR spectroscopy. First, the ν(Pt–H) absorption bands are usually very weak, due to the low dipole moment involved in the Pt–H bond. Second, in most of the cases, only linear Pt–H species can be detected, since multicoordinated Pt–H species contribute in the low frequency range, which is dominated by the framework modes of the most employed support materials.

**Figure 20:**
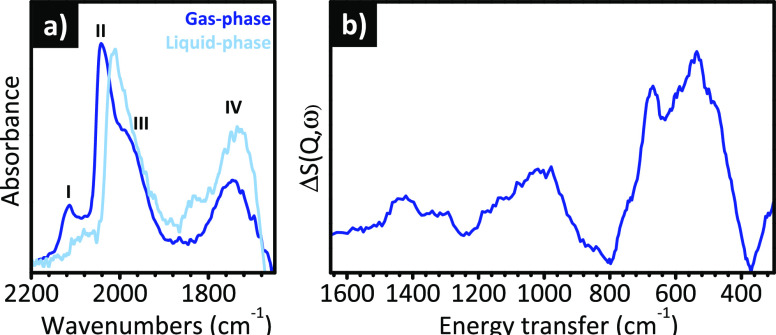
*In situ* IR spectroscopy and INS allow the identification of several types of Pt–H species on Pt NPs. (a) *In situ* IR spectrum of a freshly reduced Pt/Al_2_O_3_ catalyst in the presence of H_2_ at 70 °C either in the gas phase (10% H_2_ in N_2_, 20 mL/min) or in the liquid phase (cyclohexane as solvent). The latter spectrum has been collected in ATR-IR mode. The data reported in (a) are reproduced from ref (242) with permission from the Royal Society of Chemistry. (b) INS spectrum of H_2_ chemisorbed on the same reduced Pt/Al_2_O_3_ catalyst. H_2_ was dosed at room temperature at an equilibrium pressure of 420 mbar; the INS spectrum was measured at 20 K in the presence of H_2_. Reproduced with permission from ref (319). Copyright 2019 American Chemical Society.

Recent works by Groppo′s group demonstrate not only that the identification of Pt–H species by IR spectroscopy is feasible but also that it is possible to monitor their evolution as a function of the H_2_ coverage.^242,319,330^ The potential of the method is summarized in Figure 20a, which shows the IR spectrum of a highly dispersed, freshly reduced Pt/Al_2_O_3_ catalyst (the same shown in Figure 15b in the presence of CO) in the presence of H_2_ at 70 °C.^242^ The spectrum is characterized by four bands in the region characteristic for linear Pt–H species, at 2115, 2041, 1990, and 1740 cm^–1^, labeled as I–IV, respectively.^319^ Band I was attributed to a weak linearly adsorbed hydride,^319,324,326−328,331^ while bands II and III were attributed to strongly adsorbed linear hydride species slightly differing in the coordination geometry. Finally, band IV was assigned to linear hydrides at the interface with the support, hence interacting with the Lewis acid sites exposed at the alumina surface. As a general comment, H_2_ adsorption on Pt NPs and subnanometric clusters gives rise to a variety of linearly adsorbed species much greater than CO, and the assignment of the *v*(Pt–H) bands is less straightforward.

Moreover, as predicted by theoretical calculations, linear hydrides are not the only species formed in the presence of H_2_, also multicoordinated Pt–H species should be formed. However, these species are not detectable by IR spectroscopy, since they contribute in a frequency region dominated by the intense vibrational modes of the alumina support. In this regard, incoherent inelastic neutron scattering (INS), a complementary vibrational method extremely sensitive to hydrogenous species, appeared as a powerful technique.^332^Figure 20b shows the INS spectrum of hydrogen chemisorbed on the same Pt/Al_2_O_3_ catalyst discussed in part a. A series of different bands are observed in the 400–1200 cm^–1^ range due to Pt–H vibrations of multicoordinated Pt–H species.^333−338^ The complete assignment of these low-frequency bands can be found in ref (330). For the purpose of this Review, it is important to notice that the experimental INS spectrum was satisfactorily fitted with a linear combination of a few simulated spectra corresponding to H-covered Pt NPs having a size in the Pt_34_–Pt_55_ range (which is fully compatible with the average particle size determined by HR-TEM) and with a H/Pt ratio much larger than 1, all displaying a weak interaction with the alumina support.

Figure 20a shows also the IR spectrum of H_2_ chemisorbed on the same Pt/Al_2_O_3_ sample freshly reduced in liquid phase, with cyclohexane as the solvent. As already discussed in the case of CO adsorption, the IR spectrum of H_2_ chemisorbed in the liquid phase has the same profile of that collected in gas-phase but it is characterized by important differences in the position and relative intensity of the four bands: band I is much weaker, band II is red-shifted by ca. 25 cm^–1^, band III is almost unaffected, and band IV is presented in the same position but with a higher intensity. This indicates that, in the presence of the solvent, the relative distribution of the Pt–H species changes with respect to what found in gas-phase, likely because the overall H coverage is lower in the presence of the solvent.^242^

**Figure 21:**
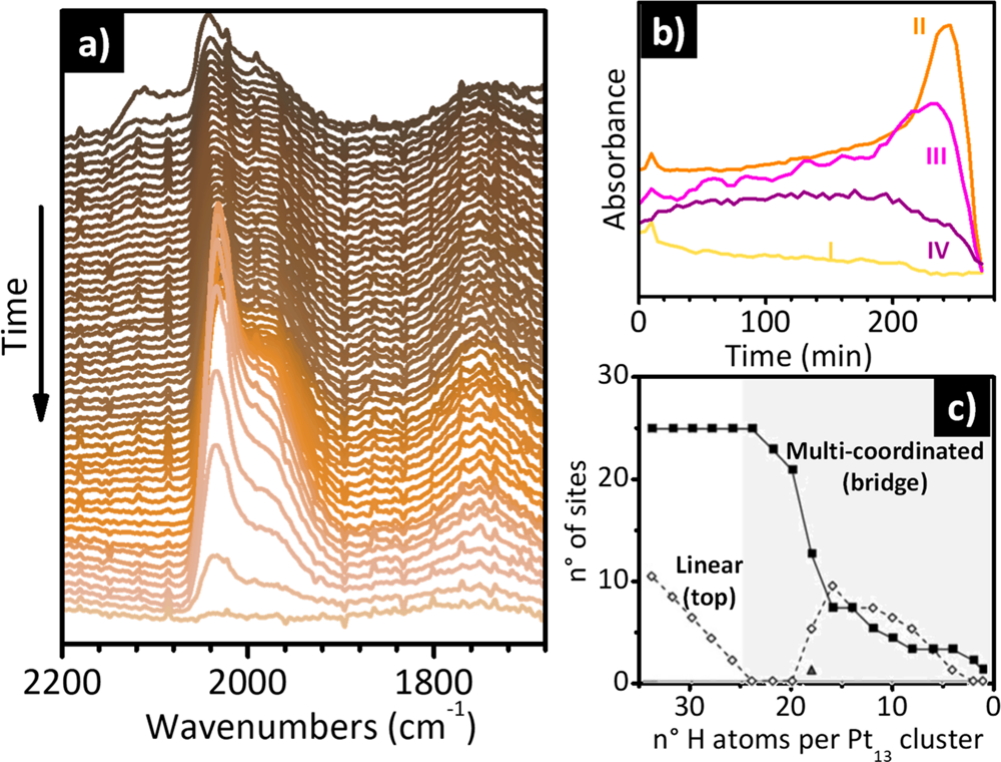
*Operando* IR spectroscopy reveals the occurrence of H_2_-induced restructuring of Pt NPs on an industrial Pt/Al_2_O_3_ catalyst. (a) Evolution of the IR spectra as a function of time (from top to bottom) for a freshy reduced Pt/Al_2_O_3_ catalyst during dehydrogenation in N_2_ flow (20 mL/min) at 120 °C. Adapted with permission from ref (319). Copyright 2019 American Chemical Society. (b) Evolution of the intensity of the bands I–IV as a function of time for the spectra shown in part a). (c) Number of linear (top, empty circles) and multicoordinated (bridge, full squares, and hollow, triangle) occupied sites for Pt_13_H_n_ models on γ-Al_2_O_3_(100). The gray background identifies the stoichiometries accessible experimentally with the Pt/Al_2_O_3_ catalyst reported in parts (a and b). Adapted with permission from ref (280). Copyright 2011 John Wiley and Sons.

Groppo’s group investigated also the evolution of the IR spectra of their hydrogenated Pt/Al_2_O_3_ catalyst upon decreasing the hydrogen coverage^319^ in order to validate the H_2_-induced Pt NPs reconstruction model predicted by theoretical calculations and summarized above.^280^Figure 21a shows the evolution of the IR spectra of the same Pt/Al_2_O_3_ sample discussed in Figure 20a upon decreasing the H_2_ coverage, while Figure 21b displays the variation in intensity of the four main bands assigned to linear Pt hydrides. Band I immediately disappears, while the other three increase in intensity for long time before rapidly decreasing and disappearing. This behavior is rather counterintuitive; in fact, the amount of detected Pt hydrides increases overtime, even though hydrogen is removed. However, these experimental data are in very good agreement with the theoretical predictions for hydrogenated Pt_13_ clusters, which are summarized in Figure 21c. At the maximum H_2_ coverage the Pt NPs are “solvated” mainly by multicoordinated hydrides, which are not visible by IR spectroscopy (but only by INS), and only a minor fraction of linear hydrides are present. Upon decreasing the H_2_ coverage, the Pt NPs undergo a progressive restructuring involving initially a decrease of both species. When the number of hydrogen atoms per each cluster is below 20, multicoordinated Pt hydrides are converted into linear ones, which are now visible by IR spectroscopy. This leads to the apparent increase in the amount of Pt hydride species detected by IR spectroscopy even though the hydrogen coverage is lower. Clearly the experimental data reported in Figure 21a,b do not match perfectly with the theoretical prediction (Figure 21c) because the particle size in the Pt/Al_2_O_3_ catalysts is slightly larger than Pt_13_ cluster and the particle size distribution broader than the theoretical one. Nevertheless, the *operando* IR data well reproduce the tendency predicted by theoretical calculation.

## 5 Summary and Perspectives

A large number of experimental and theoretical studies have demonstrated incontrovertibly that heterogeneous catalysts, exactly as their homogeneous counterparts, dynamically change under reaction conditions, *i.e.*, in the presence of adsorbates and/or at high temperatures. Recognizing the importance of adsorbate- and/or thermal-induced structural changes is fundamental not only to avoid misinterpretation in the catalyst characterization but also to rationalize the catalytic performances. The key to identifying these phenomena is to adopt *in situ* and/or *operando* characterization techniques, which allow the structural and surface changes of a catalyst to be traced as a function of time in different reaction conditions. The enormous progress made in the last 20 years in terms of instrumentation and experimental setup, has permitted to push the temporal and spatial resolution of many spectroscopic techniques and has opened the doors of the *operando* approach also to characterization techniques traditionally applied in ultra high vacuum conditions. Adopting many characterization techniques in a synergistic way to study the same catalytic system in exactly the same reaction conditions, or even simultaneously, has highlighted the occurrence of multiple types of adsorbate- and thermal-induced phenomena on a variety of heterogeneous catalysts.

Among all the spectroscopic techniques, IR spectroscopy is certainly one of the most widespread, not only in academic laboratires but also in industrial research laboratories, to such an extent that it is often considered a routine analytical technique. Nevertheless, the potential of IR spectroscopy in identifying adsorbate- and thermal-induced phenomena is often overlooked in favor of other more expensive and less available methods, such as XAS spectroscopy and high-resolution microscopy. Without detracting from these techniques, and while aware of the enormous value of a multitechnique approach, the purpose of this Review was to show that IR spectroscopy *alone* can provide relevant information in this field, provided an IR spectrophotometer and a sample environment (reaction cell, gas and/or liquid manifold, etc.) allow *in situ* or *operando* experiments to be conducted, which are two conditions easily accessible nowadays.

To start, in many of the selected case studies, the active sites structurally change under the effect of thermal treatments in different atmospheres. IR spectroscopy turns out to be one of the most sensitive methods to distinguish and quantify active sites with a different structure. This is the case of the chromium sites in the Phillips catalyst that, depending on the thermal history of the sample, can attach to chromasiloxane rings of different size, and can be more or less “exposed” to the silica surface, the two things being not necessarily correlated. IR spectroscopy of probe molecules allows the discrimination and quantification of chromium sites characterized not only by a different strain (mostly determined by the size of the chromasiloxane ring and therefore by the O–Cr–O angle) but also by a different coordinative unsaturation. Both properties are strictly related with their catalytic performance.

Additionally, metal centers in metal-substituted zeolites display a structural flexibility as a function of the thermal history of the sample. For example, in iron zeolites, thermal treatments can lead to a migration of the Fe sites outside the zeolitic framework, with the consequent creation of not only new sites grafted to the surface, which are those of greatest interest for catalysis, but also nanometric clusters of iron oxides. IR spectroscopy of probe molecules (especially NO) is one of the most sensitive techniques capable of distinguishing and quantifying the different types of Fe sites. Furthermore, the position of Fe with respect to the zeolite lattice affects the vibrational properties of the bulk in a way that is distinctive of each coordination type (inside the framework or outside). Therefore, carefully collected IR spectra in the vibrational region of the zeolite framework modes can provide direct information on the position of the Fe sites in the lattice. This potential is even more marked in the case of B-substituted zeolites, for which thermal treatments in oxidizing conditions determine an important structural rearrangement of the boron sites, which pass from a tetrahedral coordination (sp^3^ hybridization) to a trigonal planar geometry (sp^2^ hybridization), the latter associated with the formation of mildly acidic silanol groups. Again, *in situ* IR spectroscopy has the capability to demonstrate elegantly the thermally induced mobility of the B sites because of the appearance in the IR spectra of a band characteristic of the B–O vibration of these [BO_3_] units.

Finally, the thermal-induced mobility and eventual sintering of isolated Pt atoms in different atmospheres is another research field where *in situ* IR spectroscopy of probe molecules (especially CO) can offer valuable information not achievable by means of any other characterization technique. For example, IR spectroscopy of adsorbed CO is particularly suitable for differentiating Pt single sites on oxidic supports, from oxidized or partially oxidized Pt clusters.

*In situ* and *operando* IR spectroscopy also has enormous potential in revealing the occurrence of adsorbate-induced structural changes, which might have great relevance in catalysis. For example, for the Phillips catalyst, *in situ* IR spectroscopy has shown that many adsorbates, including ethylene, are capable of “extracting” the chromium sites out from the silica surface, increasing their strain. If these adsorbates do not permanently poison the catalyst, they might enhance the ethylene polymerization rate, as happens with α-olefin comonomers. Another notable example of this type is that of the titanium sites in TS-1, which are able to expand their coordination sphere in the presence of adsorbates. The structural distortion of the Ti(IV) sites is compensated by rearrangements of the zeolite framework, which can be easily monitored by *in situ* IR spectroscopy. This is considered the key to understanding the extraordinary efficiency of TS-1 as a catalyst for partial oxidation reactions in the presence of H_2_O_2_/H_2_O. Copper cations in Cu-exchanged zeolites also exhibit a flexible behavior in the presence of adsorbates. IR spectroscopy can be used to determine the degree of interaction between the cations and the zeolite lattice. In the presence of certain adsorbates (such as H_2_O and NH_3_), the Cu cations can be completely solvated and fluctuate inside the cavities and pores with a homogeneous-like behavior. In contrast, in the absence of adsorbates (*i.e.*, under vacuum), Cu cations interact with the zeolite lattice, influencing its vibrational spectrum in a different way depending on their position and oxidation state.

Finally, catalysts based on Pt NPs and SACs are extremely ductile in the presence of adsorbates, in particular CO and H_2_. *In situ* and *operando* IR measurements have been widely used to study the reconstruction of Pt NPs under different conditions, and in particular during CO oxidation reaction. Compared to other structural techniques, IR spectroscopy offers the advantage of being able to distinguish and quantify Pt sites according to their coordinative unsaturation, allowing the extent of particle reconstruction to be evaluated as a function of the experimental conditions (*e.g.*, partial pressure and/or temperature). More recently, *operando* IR spectroscopy has been applied to monitor the H_2_-induced restructuring of Pt NPs. The works summarized in this Review demonstrate not only that the identification of Pt-hydride species by IR spectroscopy is feasible but also that it is possible to monitor their evolution as a function of the reaction conditions, which is correlated to a restructuring of the Pt NPs.

In a historical period marked by the ever increasing development of extremely fast characterization methods, which unavoidably lead to the accumulation of millions of data in a single experiment that require by sophisticated analysis techniques for processing, the case-studies discussed in this Review represent a tribute to slowness and to “traditional” spectroscopy, that based on the “simple” acquisition of a few but accurate spectra. In the future we will continue to need young researchers well educated in vibrational spectroscopy, which means being able not only to conduct an experiment in the best possible conditions but also to critically observe very fine details and to correlate them with the changes that are taking place in the system. These considerations have a very general value and extend to all characterization techniques (not only spectroscopic).
